# Acceptance and expectations of healthcare workers and community during the COVID-19 vaccine rollout in Bhavnagar city, western India: a qualitative exploration

**DOI:** 10.1186/s12913-024-10885-5

**Published:** 2024-03-27

**Authors:** Mohit N. Makwana, Hiren J. Shekhda, Mihir P. Rupani

**Affiliations:** 1 Department of Community Medicine, Government Medical College Bhavnagar (Maharaja Krishnakumarsinhji Bhavnagar University), Near ST Bus Stand, Jail Road, Bhavnagar, Gujarat 364001 India; 2 Clinical Epidemiology (Division of Health Sciences), ICMR - National Institute of Occupational Health (NIOH), Indian Council of Medical Research, Meghaninagar, Ahmedabad, Gujarat 380016 India

**Keywords:** Coronavirus disease 2019 (COVID-19), Pandemic, COVID-19 vaccination, Vaccine perception, Qualitative research, Vaccine hesitancy

## Abstract

**Background:**

COVID-19 vaccine was launched in India on January 16, 2021. There is a paucity of robust evidence from qualitative studies on the acceptability and expectations of potential recipients from the COVID-19 vaccine around the time of its rollout in India. We conducted this study to explore the acceptance and expectations of the COVID-19 vaccine among the healthcare workers and community in Bhavnagar, India.

**Methods:**

During January 2021, in-depth interviews were conducted with potential vaccine recipients in Bhavnagar city—health care workers, people over the age of 50, and people of any age with comorbidities. A total of 28 in-depth interviews were performed, including 16 healthcare workers and 12 community beneficiaries. An in-depth interview guide was developed based on the constructs of the health belief model. Following written informed consent from all participants, all interviews were audio-recorded, transcribed in English and codes were generated using thematic analysis. Qualitative qualifiers were used due describe our study findings.

**Results:**

Most of healthcare workers and a majority of people from the community have shown willingness to be vaccinated with COVID-19 vaccine as they had trust on the results of the clinical trials and on the government. Some participants showed hesitancy, which was attributed to concerns regarding safety and efficacy, negative news reports in the media and lack of awareness regarding benefits of vaccine. Some healthcare workers were hesitant due to a lack of transparency in sharing the results of clinical trials. Very few showed refusal in getting vaccinated due to their belief that they won’t be infected with SARS-CoV-2 virus as they might have developed immunity against it. Most of the participants expected good efficacy, minimal/no side effects, rapid and wide coverage of vaccine and a perception of getting back to pre-COVID life sooner.

**Conclusions:**

Most of the potential recipients were willing to take the COVID-19 vaccine around the time of its initial roll out. Future vaccine rollout campaigns could reduce refusals by timely demand generation activities on vaccine safety/efficacy, involving leaders/influencers, addressing grievances, and minimizing misinformation.

**Supplementary Information:**

The online version contains supplementary material available at 10.1186/s12913-024-10885-5.

## Background

In March 2020, the World Health Organization (WHO) declared the COVID-19 disease to be pandemic, with India reporting its first case on January 30, 2020 [1, 2]. In India, the first wave of the illness peaked in the middle of September 2020 and then gradually subsided [3, 4]. India had the second wave between February and July 2021, with daily new cases exceeding 400,000 [5]. COVID-19 vaccine is regarded as the most effective means of limiting the spread of or ending the pandemic [6]. Two COVID-19 vaccines, COVISHIELD and COVAXIN, were approved for public use by the Government of India (GoI) in January 2021 [7]. On January 16, 2021, India began COVID-19 vaccination. The government scaled the vaccine program to different age groups based on vaccine availability and health system readiness.

Currently the main vaccines available in India are Oxford-AstraZeneca vaccine locally referred to as COVISHIELD, Bharat Biotech-ICMR indigenous vaccine named COVAXIN and the Russian Sputnik V vaccine that is imported [8]. Several additional vaccines, such as ZyCoV-D, COVOVAX and CORBEVAX have been given emergency use authorization by Drugs Controller General of India (DCGI), which is responsible for approving licenses for specific classes of pharmaceuticals in India, including blood and blood products, IV fluids, vaccinations, and sera [9]. Even the best vaccine would be of no use if people do not accept it.

Any vaccination campaign's effectiveness depends on the coverage and the level of acceptance. To have wide coverage and decrease hesitancy towards the vaccine, it is crucial to understand the behavioral factors of vaccine hesitancy or refusal in order to design appropriate strategies to increase vaccine acceptance [10]. A study conducted by the University of Maryland Social Data Science Center Global COVID-19 Trends and Impact Survey showed a considerable number of individuals were skeptical of vaccinations in India [11].

Several qualitative studies have investigated perception regarding acceptance of COVID-19 vaccine nationally and internationally [12–22]. Though majority of studies have been conducted abroad [12–19], their findings cannot be applied to India because of geographic, racial and sociocultural differences. Several studies have been carried out to explore the perceptions of COVID-19 vaccine among the community and health care workers in India [20–22]. These studies were either conducted before arrival of the vaccine in the market [19, 20, 22], or were conducted online [13, 14, 16, 21], or telephonically [19]. In order to identify potential obstacles to implementing a sizable vaccination campaign in India, we wanted to systematically investigate the initial responses of the beneficiaries. Our study would guide program managers in managing and mitigating any potential disappointment expressed by unmet demand for the vaccine or expectations amongst the community. Our primary objective was to explore the perceptions of healthcare workers and potential COVID-19 vaccine recipients in the community regarding acceptance and expectations from the vaccine. Secondary objective was to explore the perception of healthcare workers regarding enablers and challenges in COVID-19 vaccination.

## Methods

### Theoretical framework

This study on COVID-19 vaccination acceptance and expectations is anchored in the health belief model (HBM), a widely recognized theoretical framework for comprehending health-related behaviors, including vaccine acceptance [23]. Traditionally applied to individual health-related actions, we extended the model's utility to explore perceptions concerning COVID-19 vaccine acceptance and expectations. The HBM posits that two primary factors shape health-related behaviors: the desire to avoid illness or recover from it, and the belief that a specific health action can prevent or cure the illness [24]. Ultimately, an individual's decision is often influenced by their assessment of the benefits and drawbacks associated with engaging in health-promoting behaviors [24]. While the HBM traditionally focuses on individual health-related behaviors, we employed it to delve into perceptions regarding vaccine acceptance and expectations. We acknowledge the distinction between attitudes and actual behaviors, recognizing that our study centers on attitudes toward the COVID-19 vaccine. The HBM constructs related to the acceptance (willingness/refusal) of the COVID-19 vaccine are described below.

The term ‘perceived susceptibility’ refers to a person's own subjective perception of the risk of acquiring COVID-19. ‘Perceived severity’ refers to a person's feelings on the seriousness of contracting COVID-19 or leaving COVID-19 untreated. The term ‘perceived benefits’ refers to a person's perception of the effectiveness of COVID-19 vaccine to reduce the threat of COVID-19. The term "perceived barriers" describes how an individual feels about the challenges involved with receiving the COVID-19 vaccination, such as the possibility that it will be costly, risky (because to potential side effects), unpleasant (due to potential pain), time-consuming, or inconvenient. The stimulation that will trigger a person to receive the COVID-19 vaccine is known as a cue to action. These cues can be internal (such as a sense of moral obligation or the need to set a good example for others) or external (e.g., advice from others, illness of family member, newspaper article, etc.). The above constructs from health belief model directly affects the perceptions related to willingness or refusal towards acceptance of COVID-19 vaccine.

### Research team

The first two authors served as principal investigators for the study, while the third author provided guidance and support. The investigators are all male and have received training in qualitative research methods. The first two authors conducted all the in-depth interviews. Under the supervision of the senior (third) author, the first and second authors were pursuing their Doctor of Medicine (MD) program in Community Medicine at the time of conduction of the study. The third author possesses an MD degree in Community Medicine and worked as a teaching professor in a medical college at the time of conduction of the study. Before the commencement of the study, the authors met the study participants for building rapport, and discussed the purpose of conducting this research and the potential benefits of its findings in the future.

### Study design

We conducted in-depth interviews (constructivist paradigm) among potential vaccine recipients—healthcare workers and community (> 50 years or people of any age with comorbidities) to explore their perceptions regarding acceptance and expectations from the COVID-19 vaccine. The constructivist paradigm holds that each person develops their own worldview through their experiences and interactions with others, which means that knowledge is always being created and reconstructed as individuals interact with new experiences and viewpoints [25]. The choice of in-depth interviews, as opposed to methods like focus group discussions, was deliberate. In-depth interviews were selected to delve into individual lived experiences and perceptions, allowing participants the freedom to express their thoughts more openly. This approach contrasts with group dynamics in focus group discussions, where some individuals might feel less comfortable sharing their views or be influenced by the collective atmosphere. Importantly, our objective was not to reach a collective decision on whether to roll out the COVID-19 vaccine program. Hence, we avoided using focus group discussions, aiming instead for a more individualized exploration of perspectives. The approach (theoretical underpinning) of the in-depth interviews was an exploratory descriptive design using the theory of health belief model.

### Study setting

We conducted this study in the Bhavnagar city, which is located approximately 125 miles southwest of Gandhinagar, the state capital of Gujarat, in the western part of India. With population of ~ 0.6 million and literacy rate of 84.8%, Bhavnagar is one of the eight city of Gujarat with Municipal Corporation [26, 27]. The city belongs to one of the Tier-Y city of India [28, 29]. Bhavnagar is home to mainly service-class people with few having small scale business. All the authors were involved in COVID-19 pandemic preparedness, prevention, and management activities at a government medical college hospital in Bhavnagar city. The authors were also involved in the micro-planning of the COVID-19 vaccination sessions in the hospital and in the municipal corporation limits of Bhavnagar city. Before the COVID-19 vaccine sessions were to be initiated, the investigators noticed hesitancy not just among the community but also among healthcare workers. As a result, the research team decided to explore the perceptions among potential vaccine recipients regarding the COVID-19 vaccine in Bhavnagar city.

### COVID-19 vaccine launch in India

India’s COVID-19 vaccination strategy drew guidance from WHO’s Standard Operating Procedures, global best practices, and recommendations from the National Expert Group on Vaccine Administration for COVID-19 (NEGVAC), which comprised India’s foremost experts [30]. On January 3, 2021, ChAdOx1 nCoV-19 Corona Virus vaccine Recombinant (COVISHIELD) from M/s Serum Institute of India Pvt. Ltd. & Whole-Virion Inactivated SARS-CoV-2 Vaccine (COVAXIN) from M/s Bharat Biotech were initially approved for restricted use in emergency situation by the Subject Expert Committee of Central Drugs Standard Control Organization (CDSCO) [31]. The recommended dosages for COVISHIELD and COVAXIN were two doses separated by 12–16 weeks and 4–6 weeks, respectively [32].

The COVID-19 vaccination program in India began on January 16, 2021, initially targeting healthcare and frontline workers. Subsequently, the program prioritized vaccinating individuals aged ≥ 60 and ≥ 45 years old with comorbidities, as they were at higher risk of severe illness and death from COVID-19 [33]. From May 1, 2021, the eligible age for vaccination was expanded to include all adults above 18 years of age. The vaccine was provided for free by the government to all citizens, while private hospitals also offered the vaccine to those who could afford it [34].

### Study population and sampling

The study included healthcare workers and individuals from the community in Bhavnagar city, focusing on those aged ≥ 60 years and people with comorbidities aged ≥ 45 years. Participants who declined to take part were excluded. Purposive sampling was utilized to select individuals perceived as more knowledgeable or vocal on the topic of interest. Specifically, healthcare workers with firsthand experience during the COVID-19 pandemic were intentionally recruited from urban health centers and a tertiary care hospital in Bhavnagar city. Collaborating with urban health center personnel, we reached out to community participants, purposively selecting individuals representing diverse occupations, including senior government officers, retirees, businessmen, and laborers. This intentional selection of participants with varied backgrounds and experiences aimed to enrich the qualitative data, capturing a comprehensive spectrum of perceptions regarding the COVID-19 vaccine.

### Data collection

The in-depth interviews were conducted during January 2021. Data collection involved using an interview guide for in-depth interviews with study participants that comprised the basic demographic details. Based on the constructs of the health belief model, a detailed interview guide was prepared by the research team (Additional file 1). Their perceptions of acceptance of and expectations from the COVID-19 vaccine were the main focus of the in-depth interview guide’s questions. The interview guide of healthcare workers also focused on enablers and challenges regarding COVID-19 vaccination.

Purposefully selected study participants were approached via a phone call and the study purpose was explained to them. After scheduled appointments, participants were approached in-person for interviews as per their convenience of time and place. To ensure safety, COVID-19 safety protocols were followed. Interviews of healthcare workers were conducted at their workplaces and offices, whereas community participants’ interviews were conducted either at their workplace or at their respective religious places such as temple or mosque. Written informed consent was obtained from the study participants which was audio-recorded as part of the in-depth interviews. During the interviews, only the investigator and the participant were present. The pilot-tested interview guide was originally written in English before being translated into Gujarati, the primary language in Gujarat.

The interviews were conducted till saturation of response was achieved. After each interview, important points were noted down which were then compared in subsequent interviews to confirm saturation. Once saturation was believed to be achieved, two more interviews were conducted to confirm it. Interviews lasted 16 min on average (ranging from 12–20 min). A total of 28 in-depth interviews were conducted to achieve theoretical saturation.

Each in-depth interview began with formal interactions about the COVID-19 pandemic, their work and other demographic details. Only one in-depth interview each day was conducted to preserve the quality of the data. With prior written informed consent from each study participant, all interviews were audio recorded. No repeat interview was conducted. The study participants did not get the transcripts or analysis in order to offer corrections or comments.

### Analysis

The original Gujarati audio recordings were transcribed into English using Microsoft Word (Additional file 2). The coding process involved collaborative use of the ‘comment’ feature in Microsoft Word by the first two authors, with subsequent reconciliation by the third author. Coded information was then organized in a Microsoft Excel document. Thematic analysis, rooted in the health belief model, guided the categorization of codes into themes, employing an inductive approach driven by our data. This methodology revealed underlying patterns and themes within the qualitative data, allowing for a comprehensive exploration of participants' perspectives on vaccine acceptance. Thematic analysis was complemented by content analysis, which enriched the qualitative exploration. The integration of content analysis included the application of qualitative qualifiers, introduced during a qualitative research methods workshop at our institute (Table 1). Despite exhaustive efforts, the source of these qualifiers, including exploration of various textbooks, remains unidentified. It is worth noting that these qualifiers appear to align with established qualitative data analysis techniques [35–37]. These qualitative qualifiers played a pivotal role in providing a semi-quantitative description of findings, facilitating the nuanced assessment of opinions across the dataset. This comprehensive approach, blending thematic and content analyses, underscores the rigor of our methodology, offering a robust foundation for the nuanced exploration of perceptions surrounding vaccine acceptance.

**Table 1:** Qualitative qualifiers for semi-quantitative aspects of our analysis

Proportion of respondents	Qualitative qualifiers	Description
< 10%	< 1 +	Very few
11–24%	1 +	Some
25–49%	2 +	Approximately half
50–74%	3 +	Majority / over half
75–89%	4 +	Most
> = 90%	5 +	Almost all

## Results

We enrolled 19 healthcare workers and 16 community participants of Bhavnagar city (Gujarat state, western part of India) for our study, out of whom three healthcare workers and four community participants refused participation without citing any reasons. Thus, a total of 28 in-depth interviews were conducted, including 16 healthcare workers and 12 community participants. The median (interquartile range IQR) age of the participants was 38 (28–56) years. Among the healthcare workers, 11 were doctors and 5 were the nursing staff. We have analyzed the perceptions of acceptance of COVID-19 vaccine among its potential recipients from community and healthcare workers. We also described enablers and challenges which play a major role in vaccination coverage based on the perceptions of the healthcare workers.

### Acceptance of COVID-19 vaccine among potential vaccine recipients

**Fig. 1:**
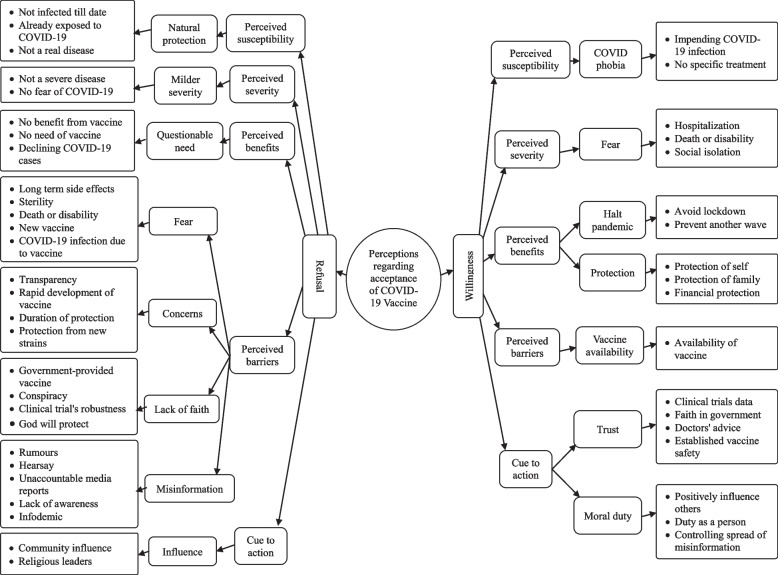
Perceptions of healthcare workers and community regarding acceptance of COVID-19 vaccine in Bhavnagar during January 2021

Theme acceptance was described by sub themes willingness and refusal to receive COVID-19 vaccine (Fig. 1). Willingness and refusal sub themes were categorized and coded based on the theoretical frame work of health belief model (refer Additional file 3 for description of each code).

#### Willingness for COVID-19 vaccine

Perception of participants regarding willingness to receive COVID-19 vaccine were categorized based on constructs of theory health belief model i.e., perceived susceptibility, perceived severity, perceived barriers, perceived benefits, cue to action. Most of healthcare workers and a majority of people from the community have shown willingness to be vaccinated with COVID-19 vaccine.

#### Perceived susceptibility

Perceived susceptibility is an individual's subjective perception of the risk of contracting COVID-19. There is a wide range of feelings of personal vulnerability to the virus, which plays a major role in determining vaccine acceptance. Acceptance of the vaccine is likely to increase when people are fearful of becoming infected with COVID-19 and the potential negative impacts it could have on their health and finances.

*“It is for our safety; I understand that much. We have to go out and the vaccine can give us the protection of some level and keep us safe from corona”* (Male, Community Participant 01).

#### Perceived severity

The perceived severity of COVID-19 is a major factor in vaccine acceptance. People often take into account the medical and social consequences of the virus, such as death, disability and disruption to family life and social relationships, when assessing its severity. The belief that COVID-19 can lead to hospitalization, disability, or social isolation has been a driving force in increasing acceptance of the vaccine.

*“It is good if we take this vaccine as cases of corona are increasing and many people have died too, we get immunity against corona by taking the vaccine. So, it is necessary to take vaccine”* (Female, Healthcare worker 05).

#### Perceived benefits

Perception of the ability of vaccines to lessen the threat posed by COVID-19 is a key factor in determining whether or not a person will accept the vaccine. It is believed that vaccines against COVID-19 can not only prevent another wave of cases, but also protect individuals and their families from infection, while also allowing for the lifting of lockdown and movement restrictions, which would lead to an improvement in the economy.

*“Vaccine can be a thread to hold on in difficult times, the whole world is fighting corona, to get rid of it we have to take vaccine.”* ( Male, Healthcare worker 08).

*“At present, if we take corona vaccine then our nation will be free completely free from corona disease and our GDP will rise in comparison to other countries.”* (Male, Community participant 08).

*“Everyone should take COVID-19 vaccine which is necessary. Though they have suffered from or not, If the vaccine is taken then chances of getting COVID-19 infection would be decreased in future.”* (Male, Healthcare worker 13).

*“In COVID-19 Pandemic, when there is no specific medicine, Vaccination is the only salvage. That's why vaccination is good and it has to be done.”* (Male, Healthcare worker 09).

#### Perceived barriers

People's feelings about the obstacles to getting vaccinated against COVID-19 are varied. The most commonly cited barrier for those eager to receive the vaccine is its availability. Unfortunately, due to limited supplies and high demand, many people have been unable to access the vaccine. This has caused frustration and anxiety among those who are eager to protect themselves and their loved ones from the virus.

*“Mass production of vaccine should be done and along with government facility, vaccine should be made available in private setups as well. So, people, who are capable, can take vaccine earlier at their own expense.”* (Male, Community participant 03).

#### Cue to action

This stimulation is required to nudge the decision-making process toward accepting a recommended health intervention. Factors such as trust in vaccine/clinical trials/government/doctors, the unavailability of specific treatments for COVID-19, the safety of vaccinations for healthcare workers, the free cost of the vaccine, and the duty of individuals to positively influence others, all act as a stimulus in increasing the willingness to take the COVID-19 vaccine.

*“After explanation and witnessing the condition and knowing how’s the situation of beneficiaries (Health care Workers) who have vaccinated in initial phase, all will immediately accept it too.”* (Male, Healthcare worker 09).

*“Why Doctors are taking vaccine? Because they have faith in it. For example, an owner is eating at his own restaurant because he has faith in it. Similarly, doctors are getting vaccinated, because they are aware of vaccines. So, if doctors are taking vaccine, we should take vaccine.”* (Male, Community participant 07).

#### Refusal for COVID-19 vaccine

Perception of participants regarding refusal to receive COVID-19 vaccine were categorized based on the constructs of HBM i.e., perceived susceptibility, perceived severity, perceived barriers, perceived benefits, cue to action. Some participants showed hesitancy and very few showed complete refusal in getting vaccinated.

#### Perceived susceptibility

People's subjective assessment of the risk of obtaining COVID-19 has led to vaccine hesitancy, which is defined as "the delay in acceptance or refusal of vaccination despite the availability of vaccination services". Reasons for this hesitancy can include a lack of fear of COVID-19 infection, not having been infected to date, having already been exposed to the virus, declining COVID-19 cases, and feeling no need for the vaccine. All of these factors contribute to the refusal of getting vaccinated against COVID-19.

*“I do not feel personally that I need it and it will cure me. There is a decline in cases in Gujarat and in Bhavnagar. So it’s okay I don’t get vaccine.”* (Male, Community participant 12).

*“For 1 year, I have been in contact with COVID-19 patients. So I would already have antibodies which will work as a vaccine for me. So there is no need to take a vaccine.”* (Male, Healthcare worker 08).

*“Many people are thinking that there is no disease like corona so they don’t want to take vaccine. Many people are saying that they have already developed antibody against it, so they are avoiding it.”* (Male, Healthcare worker 13).

#### Perceived severity

The perceived severity of COVID-19 plays a major role in vaccine acceptance at large. Since many people with COVID-19 experience only mild symptoms or none at all, it can be difficult to accurately assess its risk. The belief is that COVID-19 infection is not severe enough, as they have already been infected with COVID-19 and recovered without any major complications.

*“If we get infected with Corona, we can get cured by treatment. Even I have suffered from corona and have recovered.”* (Male, Community participant 04).

*“I have been working in hospital as well as field and have already came in contact with many corona positive patients. Till date, I have no symptoms of corona.”* (Male, Healthcare worker 03).

#### Perceived barriers

Perceived obstacles in getting vaccinated against COVID-19 such as Fear, Concerns, Mistrust or Lack of faith and Misinformation account for refusal.

##### Fear

People are scared of what they don't understand, and while there has been a tremendous amount of effort put into outlining the safety and efficacy of currently available vaccines, still many remain unconvinced. Fear can be incredibly powerful in that it's hard to logic your way out of something when you are pushed into unfamiliar territory or overwhelmed by fear for the unknown. Fear plays a driving force against acceptance of the COVID-19 vaccine leading to hesitancy or refusal which is attributed to Fear of a new vaccine, Fear of side effects, Fear of disability, sterility or even death and Fear of COVID-19 infection due to the vaccine. Both disease and vaccination may be associated with some risk. In absence of disease, fear of disease has been replaced by fear of vaccine side effects for some people. Approximately one-half of community members and very few of health care worker were having one or another fear of taking the vaccine.

*“I don’t have any fear of Corona. But I am having fear of taking vaccine because of side effects.”* (Male, Community participant 04).

*“People want to live; they don’t want to die by taking the vaccine. By taking vaccine various parts of body are affected like kidney”* (Male, Community participant 11).

*“We cannot say with 100% surety that there won’t be any side effects. There might be some major or minor side effects. So, I don't believe that I can take the vaccine.”* (Female, Healthcare worker 06).

*“Everyone is in fear that if this vaccine will cause any serious side effect on them than what they will do? Common side effects are ok but if serious side effect occurs than we would like to be COVID positive rather than having serious side effects.”* (Female, Healthcare worker 10).

##### Concerns

During the ongoing COVID-19 pandemic, many individuals have expressed doubts and concerns about the speedy development of new vaccines. People think that vaccine development has been rushed and there is a lack of transparency in the results of clinical trials which is hampering their confidence. Parallel production and testing are seen as concerning rather than a good thing. The primary concern stems from the belief that speed does not give enough time to see efficacy, long-term side effects, duration of protection and protection against new strains. Due to this the Attitude of wait and watch was noted. Approximately one-half of community members and some of the health care workers have concerns surrounding the vaccine and its effects.

*“According to me, we should ‘Wait and Watch’. After a couple of tests and trials on people and observing side effects and such things, I will decide about it.”* (Male, Community participant 05).

*“I am not having enough evidence that we should take the vaccine. I have no confidence in the vaccine as the government has kept details of vaccine trials confidential.”* (Male, Healthcare worker 08).

*“If I take the vaccine and anything happens to me, who will take responsibility for my family.”* (Female, Healthcare worker 03).

*“Many health care workers are discussing that after taking vaccine if we would develop anything (Complication) than what would happen to our family?”* (Female, Healthcare worker 10).

##### Mistrust/Lack of faith

There is still widespread mistrust of COVID-19 vaccinations in many communities. This mistrust can be due to a broad range of reasons that range from culture and beliefs, healthcare access disparities and distrust of government or corporate power. Mistrust is rooted because of the inconsistent information about COVID-19 as a whole and the perceptions that the vaccine development and clinical trials are all being influenced by political agendas. A part of the community believed that religious faith is the only true defense against COVID-19, therefore vaccination is pointless. Some of the community members and very few health care workers were having mistrust in the vaccine.

*“All Big leaders have not got vaccinated and they are telling people to get vaccinated. Prime ministers of China and Russia have got vaccinated and over here they have not taken.”* (Male, Community participant 12).

*“I have heard that the Government is planning to kill everyone. I am not interested in getting vaccinated.”* (Male, Community participant 11).

*“Every-day in WhatsApp there is news that a common man has died after taking the vaccine, in the case of corona people can recover by taking treatment but in the case of vaccine, people die due to side effects. No person wants to die.”* (Male, Community participant04).

*“India’s vaccine has not completed all tests from which third phase trial is not yet completed.”* (Male, Community participant 12).

##### Misinformation

Along with the COVID-19 pandemic, Easy access to information due to internet connectivity and electronic media has ignited ‘Infodemic’ leading to a set of challenges with a magnitude that was not encountered before. Misinformation, whether it be rampant conspiracy theories or simply ill-informed opinions, is often propagated through unreliable sources and can lead to misconceptions about the safety and efficacy of vaccines. False news, unaccountable media reports, conspiracy theories and propaganda are being shared at the speed of light, increasing vaccine refusal. Due to lack of awareness, judgments are based on hearsay rather than evidence.

*“Mediums like WhatsApp are present. More rumors are spread through it.”* (Male, Community participant 05).

*“At present media has advanced a lot like WhatsApp and news channel, such that news reaches us first even before it has happened. Recently my relative took the vaccine and his lower body part got paralyzed.”* (Male, Community participant 04).

*“I think proper awareness we required is not there in the general public. The main reason is negative news from newspapers or hearsay.”* (Male, Healthcare worker 09).

*“No one has trust in vaccination as all are spreading rumors. So, I do not have interest in it. Due to all rumors present, I have fear due to it.”* (Male, Community participant 11).

#### Perceived benefits

Perception of the vaccine’s effectiveness in reducing COVID-19 threat is a contentious issue. Vaccination does not confer complete immunity and infection can still occur in vaccine recipients. Many people believe that the vaccine is not effective and does not reduce the risk of COVID-19 infection, which is contributing to the vaccine refusal.

*“I do not feel any role of it (vaccine) in ending the pandemic.”* (Male, Community participant 10).

*“There is no country in the world which has defeated corona by using the COVID vaccine, so in our country, I do not feel like we can be corona free with COVID vaccine.”* (Male, Community participant 04).

*“At Present, I am not willing to get vaccinated. Personally, I do not feel that I need it and it will cure me.”* (Male, Community participant 12).

#### Cue to action

This stimulus is required to start the decision-making process in order to embrace a recommended health action. To prevent refusal, influential figures such as religious and political leaders, actors, and athletes should be utilized as the face of vaccination drives by getting vaccinated first. Building trust through activities like strengthening grievance redressal mechanisms and addressing fears or misconceptions by minimizing misinformation, and disseminating factual and positive news through more IEC in all media formats, can help to mitigate refusal.

*“Wide publicity and awareness campaign (is required.) As I told you Celebrities and leaders should take vaccine than there will be more benefit. So, people will get motivation that they should get vaccinated. Neutral People like NGOs or doctors like you give awareness than it would create difference in mindset of people.”* (Male, Community participant 12).

*“When my family doctor will say, my family, my community will have trust and they will say that vaccine is needed to be taken and if it is necessary than I am ready for it.”* (Male, Community participant 11).

*“In social media, rumors are more exaggerated than reality. Benefits of vaccine is less present there. So Good Publicity should be done in counter with false publicity. There should be news of individual who have taken vaccine and there are no side effects.”* (Male, Community participant 07).

The identified drivers for vaccine willingness were faith in vaccination’s perceived safety, free vaccines, a sense of national duty towards eradicating COVID-19, perceived limited or no risk in vaccination, access to trustworthy information on the COVID-19 vaccine, and easy availability of the vaccine through hospital personnel.

Conversely, the concerns contributing to vaccine refusal included less active COVID-19 cases, a preference for natural immunity, misinformation about the vaccine, a lack of perceived safety, concern about adverse effects, fear of faulty or fake vaccines, and government conspiracy theories.

### Expectations from COVID-19 vaccine among potential vaccine recipients

**Fig. 2:**
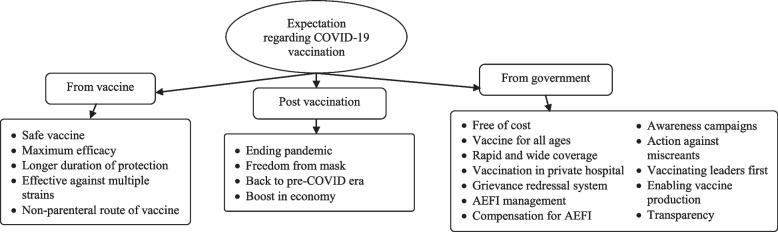
Perceptions of community on expectations from COVID-19 vaccination program in Bhavnagar during January 2021

Participants mentioned a number of expectations ranging from expectations from government, expectations from vaccine and expectations post vaccination (Fig. 2, refer Additional file 3 for description of each code).

#### Expectations from government

The global COVID-19 pandemic has had a dramatic and far-reaching impact on people's lives, as well as on the economy. The major expectation from governments were as follow: free of cost, vaccine for all age group, rapid and wide coverage, vaccination in a private hospital, grievance redressal system, AEFI management, compensation for AEFI, awareness campaigns, action against people spreading misinformation, vaccinating leaders first, favorable atmosphere for vaccine production, transparency.

*“Maximum vaccination coverage should be done in at least people with comorbidity and people aged more than 50 years.”* (Male, Healthcare worker 09).

*“Government should make this vaccine available to each and every citizen of India as soon as possible.”* (Male, Healthcare worker 01).

*“All Health care workers are going to be vaccinated and if any staff member develops any serious side effects, there should be provision of compensation. If any of them is admitted in hospital, it’s expense should be paid by government and not by the staff themselves.”* (Female, Healthcare worker 11).

#### Expectations from vaccine

As the development of a vaccine for COVID-19 presents itself as a viable option, people around the world have many expectations such as vaccine with minimum or no side effects and maximum efficacy, longer duration of protections, effective against multiple strains and non-parenteral route.

*“Vaccine should be long term effective and it should have maximum efficacy.”* (Male, Healthcare worker 08).

*“According to me, whichever vaccine we take we should be assured that for 6 months or 2 years, we would not get infected. Government should provide assurance certificate which we will get protection from infection for a period of time.”* (Female, Community participant 06).

#### Expectations post vaccination

With the arrival of vaccines against COVID-19, people began to anticipate a shift in their lifestyle post-vaccination such as freedom from mask, getting back to Pre-COVID life, ending the pandemic and boosting the economy.

*“If an effective vaccine is introduced then it (COVID-19) would get minimized. As measles was controlled in the past, the same way this disease should be controlled. So, people can get rid of wearing masks which is an expectation I am having.”* (Male, Community participant 05).

*“After COVID vaccination, all precautions should be present except mask. Mask is a little bit of problem.”* (Male, Community participant 07).

*“Vaccination will play a major role in ending COVID-19 Pandemic. 100 percentage. As we look into results of trials, it seems we will get complete success in vaccination.”* (Male, Healthcare worker 09).

### Factors influencing COVID-19 vaccination coverage

**Fig. 3:**
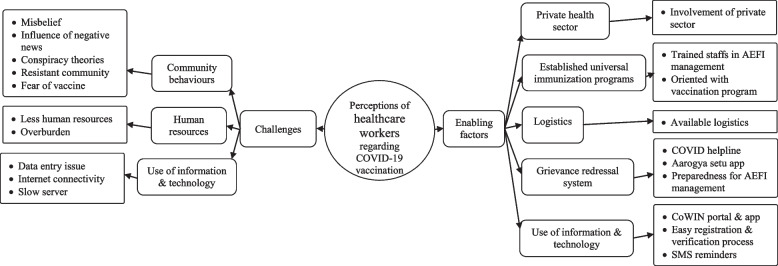
Perceptions of healthcare workers regarding challenges and enabling factors related to COVID-19 vaccination program in Bhavnagar during January 2021

Healthcare workers mentioned several factors influencing vaccination coverage that affects coverage directly i.e., increasing coverage as well as decreasing coverage and these were categorized as challenges and enabling factors (Fig. 3, refer Additional file 3 for description of each code).

#### Challenges

From the view of healthcare workers, the Main challenges were limited human resources, issues related to IT and community behavior. Community behavior seems the biggest challenge, which accounts for fear of vaccines, misbelief, the influence of negative news, conspiracy theories surrounding COVID-19 vaccine and vaccine resistant community. Constraints in Human resources were noted due to already fewer numbers of health personnel and parallel routine health activities along with COVID-19 vaccination sessions. Slow server and internet connectivity issues were the main IT-related challenges.

#### Enabling factors

India is successfully conducting universal immunization programs for years. An established immunization system, already oriented and trained staff for vaccination and AEFI management is one of the biggest enabling factors in COVID-19 vaccination program. Integration of IT by creating CoWIN app and portal made vaccination registration and verification process much easier. For grievance redressal, COVID helpline number, Aarogya setu app and AEFI management centres were set up. Along with sufficient logistics for vaccination, the involvement of private health sector was an important enabling factor.

## Discussion

India began its national vaccination campaign against COVID-19 on January 16, 2021. The program prioritized health care workers, frontline workers, people over the age of 60 years and those over age of 45 years having certain comorbidities [34]. The present study explore the qualitatively the reasons and meanings behind acceptance and expectations from the COVID-19 vaccine among its potential recipients.

### Summary of main findings

We found that potential recipients who showed willingness for COVID-19 vaccine perceived benefits that vaccine will provide protection and prevent another lockdown. COVID-19 was perceived as a severe disease, leading to fear of isolation, hospitalization or death. Trust and feeling of moral duty acted as motivators for vaccination. Fear of side effects of newer COVID-19 vaccine, lack of trust, misinformation and concerns regarding vaccine safety and efficacy were barriers among people refusing to be vaccinated. Perception of lower or no susceptibility to COVID-19 led to vaccine refusal. Prime challenge was fear, which was fueled by misinformation on safety & purpose of vaccine leading to distrust and ultimately refusal.

### Acceptance and expectations of COVID-19 vaccination

These findings affirmed our assumptions of willingness and refusal to COVID-19 vaccine linked to the health belief model [24]. A Canadian study revealed that the top worries of those who were reluctant to embrace the COVID-19 vaccine were: concerns about risks, perceived safety and side effects of vaccination, which supports the findings of present study [38].

‘Infodemic’ term, highlighted by the WHO, described the unfounded information, which does not reflect the truth [39]. It includes panic news, rumors, and conspiracy theories aimed only at misleading society. Humans are prone to believing and passing on alarming information. Unfortunately, people have to rely on the search engine and hope that it will show the most truthful and complete information. Most people in a fast-paced world have neither the time nor the energy to confirm the authenticity of the information. Similar to a study conducted in USA, present study found that participants encountered a range of misinformation, usually through news, social media sources and hearsay [40]. With instant messaging apps, the user has the option to not only forward a message to others but also create a new message while referencing the original information as "heard" or "it was said that…" As a result, the original message is further distorted and overgrown with rumors [41]. Strong "pro-vaccine" advertising may possibly be having the opposite impact on some people, increasing their reluctance.. Over-communicating about events that are minor or might not be related to vaccination may create unnecessary public concern and needlessly damage public confidence [42]. Negative information generally receives more attention than positive information, from evolutionary standpoint, paying attention to negative information could be crucial for survival, while missing positive information does not usually constitute the same threat [43].

Current study found that people believed that they don’t need vaccine as they are already using different measures of prevention such as mask and social distance. In order to avoid COVID-19 infection, the community should be aware that personal protective equipment cannot replace immunization [44]. The facts and perceptions around the COVID-19 vaccination have a significant impact on the public's readiness to accept the vaccine, which is not constant [45]. A study conducted in china corroborates with current study findings that the number of daily confirmed cases and capability of healthcare facilities in a certain area may have an impact on the vaccine's acceptance [46].

Study showed that people expected, transparency in vaccine trials to gain trust in vaccine; free, safe and effective vaccine with minimum/no side effects; proper management of AEFI with appropriate compensation; post vaccination mask free life. Working with people and communities to meet these expectations is crucial. Rather than belittling or rejecting their worries, engaging in a respectful conversation with them is essential [47]. To boost vaccination adoption, it is also crucial to make sure that the general public and healthcare professionals have access to credible and sufficient information about vaccines.

In addition to ideology and trust, other socio-demographic factors also affect views about vaccination. Age and income, in particular, appear to have an impact on how people choose which vaccines to receive [46]. Lifelong immunity after COVID-19 vaccination is unlikely and people would have to take subsequent booster doses at regular intervals [48]. When compared to more recently immunized individuals, at least one cohort of individuals with older vaccination records experienced more breakthrough infections [49]. In our study, few people perceived that COVID appropriate behavior is a better option as compared to COVID-19 vaccination in the prevention of disease. People appeared to think that following through on these safety measures would be sufficient to stop this epidemic. A report by WHO stated that depending on one's point of view, belief in the effectiveness and safety of the vaccines was a significant facilitator or obstacle. In terms of participants' reported vaccine reluctance and refusal, more people expressed concerns about the vaccine's efficacy and safety than they did belief that it is unnecessary [42]. With high levels of vaccine hesitancy, population seems to be more easily affected by misperceptions about vaccines.

OECD (The Organization for Economic Cooperation and Development) policy responses to COVID-19 mentioned that the government should take the lead in contacting the media to get the information right and accurate [50] in order to address any concerns related to vaccine (safety, efficacy, availability etc.), individual/community related issues (beliefs, knowledge, health related behaviors and mistrust etc.) and contextual issues (historical influences, conspiracy theories and non-transparency etc.), which are consistent with the findings of present study [51]. Besides, Celebrities can help increase people's willingness and motivation to become immunized by channeling the right messages through local community leaders.

### Challenges and enablers of COVID-19 vaccination

The findings of our study sheds light on challenges and enabling factors influencing COVID-19 vaccination coverage providing valuable insights into the complexities of vaccine rollout efforts. One of the main challenges highlighted by healthcare workers is the significant impact of community behavior on vaccination coverage. This aligns with previous research indicating that vaccine hesitancy, fuelled by fear, misbelief, and misinformation, presents a substantial barrier to achieving high vaccination rates [52, 53]. The influence of negative news and conspiracy theories surrounding COVID-19 vaccines further exacerbates this challenge, echoing findings from studies on vaccine hesitancy during previous outbreaks [54, 55].

Limited human resources emerge as another significant challenge, consistent with research indicating that healthcare workforce shortages can hinder vaccine delivery and administration [56, 57]. This challenge is compounded by the need to balance COVID-19 vaccination efforts with existing routine healthcare activities, reflecting the strain on healthcare systems during pandemics [58].

IT-related challenges, including slow server and internet connectivity issues, are also identified as barriers to vaccination coverage. This underscores the importance of robust technological infrastructure in facilitating efficient vaccine rollout, as highlighted in studies emphasizing the role of technology in enhancing healthcare delivery. Kolff et al. [59] pite these challenges, several enabling factors contribute to the success of COVID-19 vaccination efforts in India. The well-established universal immunization program, with its trained staff and infrastructure for vaccination and adverse event management, serves as a cornerstone of the vaccination program [8]. The integration of IT, exemplified by the CoWIN app and portal, streamlines the vaccination registration and verification process, aligning with research advocating for the use of technology to improve immunization coverage [59].

While logistics in COVID vaccination, inadequate infrastructure and vaccine shelf life concerns can be a challenge [60]. our research identified the available logistics chain as a crucial enabler for COVID-19 vaccination.

Moreover, the establishment of grievance redressal mechanisms, such as COVID helpline numbers and AEFI management centers, reflects proactive efforts to address concerns and ensure public confidence in the vaccination program [30]. The involvement of the private health sector and sufficient logistics for vaccination further bolster the vaccination program's effectiveness, highlighting the importance of public–private partnerships in healthcare delivery [61].

### Future implications

Our research was conducted during the initial phase of COVID-19 vaccine rollout in India, and its implications extend to future vaccination campaigns, including the introduction of upcoming HPV and adult/adolescent BCG vaccines [62–66]. Our study highlights the critical importance of addressing vaccine hesitancy through transparent communication, community engagement, and technology integration is key to enhancing vaccine uptake. Involving influencers, strengthening grievance redressal mechanisms, and monitoring misinformation on social media are vital for bolstering public confidence. By implementing targeted interventions informed by our findings, health authorities can improve vaccine acceptance and uptake, ultimately contributing to controlling the spread of diseases and mitigating its impact on public health.

### Strengths and limitations

An in-depth understanding of the topic under investigation was provided by the comparison and contrast of participant views, which was accomplished by purposive sampling of participants that includes community members and health care workers with a range of demographic and socioeconomic characteristics (gender, age, and occupation). The present study is reported as per the COREQ guidelines (Additional file 4) [67]. The study setting was limited to inly Bhavnagar city, so findings of the study can only be generalized to the Tier-Y cities similar to Bhavnagar. While the health belief model traditionally focuses on individual health-related behaviors, we have expanded its application to explore perceptions regarding vaccine acceptance and expectations. However, we acknowledge that this extension represents a limitation of our study.

## Conclusions

Despite mass vaccination efforts and widespread awareness campaigns, it is probable that many individuals will still be hesitant to receive the vaccine, particularly during the early stages of a new vaccine's rollout during a pandemic. Integration of Information Technology (IT) in vaccine roll out made the whole process faster and easier. To increase the acceptance and avert refusal, influencers such as religious & political leaders, actors, sportsperson, etc. should be involved as a flag bearer of vaccination drives by being vaccinated first. Trust building activities like strengthening grievance redressal mechanisms and addressing fears or misbelief by minimizing misinformation, spreading factual & positive news by more Information, Education and Communication (IEC) in all media formats are recommended. Considering the massive scale of social media usage for information sharing, there has to be systematic monitoring of the circulation of misinformation on it. The results of this study can also be applied to the creation of a survey tool for future surveys to determine the public's perception of the future newer vaccine rollout.

### Supplementary Information

**Supplementary Material 1.**

**Supplementary Material 2.**

**Supplementary Material 3.**

**Supplementary Material 4.**

## Data Availability

All data generated or analyzed during this study are included in this published article [and its supplementary information files].

## Abbreviations

COVID-19: Corona Virus Disease 19
FGD: Focus Group Discussion
NEGVAC: National Expert Group on Vaccine Administration for COVID-19
WHO: World Health Organization
CDSCO: Committee of Central Drugs Standard Control Organization
AEFI: Adverse Event Following Immunization
IEC: Information, Education and Communication
COREQ: Consolidated criteria for Reporting Qualitative research
ANM: Auxiliary Nurse and Midwife
GNM: General Nursing and Midwifery
