# Supplementary material for: Acceptance and expectations of healthcare workers and community during the COVID-19 vaccine rollout in Bhavnagar city, western India: a qualitative exploration
Source: BMC Health Serv Res. 2024 Mar 27;24:386. doi: 10.1186/s12913-024-10885-5 (PMC10976747; doi:10.1186/s12913-024-10885-5)
Supplement: Supplementary file 1 — Supplementary Material 1. [file 12913_2024_10885_MOESM1_ESM.docx]

**Interview guide: Healthcare Workers**

**Purpose of interview:**

Good morning/evening, I am Dr. Mohit Makwana/Dr. Hiren Shekhda working as resident doctor in Govt. medical college, Bhavnagar. As it has been explained to you, we are here to explore your perception regarding COVID-19 vaccine.

The research question to be answered is **“Among potential recipients of COVID-19 vaccine, what are the perceptions regarding acceptance and expectations from the vaccine?”**

**Confidentiality**

All information collected about you during the course of the research will be kept strictly confidential. Data will not be shared with anyone outside of the study.

**Consent: Are you willing to participant in study?** Can we record audio of this interview? Please sign inform written consent form to participate in study.

**Briefing/introduction**:

In this dark era of COVID-19 pandemic, vaccine seems to be the only ray of hope as no specific treatment is available. The scientific fraternity started working on the vaccine and the whole world was eagerly waiting for the vaccine. Now that the vaccine is ready, we are here to discuss what are the perceptions regarding acceptance and expectations from the vaccine among its potential recipients.

It is expected that you feel free to express your opinion. All the information will be kept confidential and anything you say will be not linked to your names and it will not be disclosed to anyone, so feel free to give your comments and your detailed opinion rather than saying just yes or no.

**Opening /Ice breaking question:**

1. What is your name? (In consent form)
2. What is your age?
3. What is your occupation?
4. Have you or any of your family members have been infected with Covid-19?

**Specific questions:**

1. What is your opinion on getting vaccinated against COVID-19 in the current scenario?
   - 1. What are the reasons for your answer (Refusal/ Willingness/ Hesitancy/ Eagerness)?
     2. What is your opinion on the concern surrounding the side effects related to COVID-19 vaccine?
     3. What is your opinion on any misinformation affecting the acceptance of Covid-19 vaccine among the community?
2. What are your expectations from the vaccine? (take cues from the answers above)
3. What do you think the government should do to meet your expectations?
   - - - 1. What do you think, when you will be confident/willing to get vaccinated? (What changes can be made in the process?)
4. What is your opinion on compliance to COVID-appropriate behaviour post-vaccination among us?
5. What is your opinion on the role of COVID-19 vaccine in ending the pandemic?
6. What do you think are the Challenging factors of COVID-19 vaccination program?
7. What is your opinion on availability of Human Resources in COVID-19 vaccination program?
8. What is your opinion on role of Logistics in COVID-19 vaccination program?
9. What is your opinion on role of Community perceptions and Behaviours in COVID-19 vaccination program?
10. What is your opinion on current grievance redressal for COVID-19 vaccination program?
11. What do you think are the Enablers of COVID-19 vaccination program?
12. What is your opinion on role of existing mechanism under Universal Immunisation Programme (UIP) in COVID-19 vaccination program?
13. What is your opinion on role of IT (Information Technology) in COVID-19 vaccination program?

**Probing of question if needed.**

Can you elaborate that… please explain… anything else…?

**Exit question:**

**We are about to closed interview …**

Is there anything more you want to add that you were not able to say regarding acceptance and expectations of COVID-19 vaccine in this interview?

**De-briefing:**

During this interview, a few aspects on acceptance/ refusal/ hesitancy that you mentioned were ………… and some of your expectations were………………

**I thank you for participating actively in the interview.**

Can I call you for knowing any detail regarding this if required?

**Meanwhile facilitator observer and recorder check note, and audio recording.**

Same day, possible codes will be enlisted as far as possible.

**Interview guide: Community**

**Purpose of interview:**

Good morning/evening, I am Dr. Mohit Makwana/Dr. Hiren Shekhda working as resident doctor in Govt. medical college, Bhavnagar. As it has been explained to you, we are here to explore your perception regarding COVID-19 vaccine.

The research question to be answered is **“Among potential recipients of COVID-19 vaccine, what are the perceptions regarding acceptance and expectations from the vaccine?”**

**Confidentiality**

All information collected about you during the course of the research will be kept strictly confidential. Data will not be shared with anyone outside of the study.

**Consent: Are you willing to participant in study?** Can we record audio of this interview? Please sign inform written consent form to participate in study.

**Briefing/introduction**:

In this dark era of COVID-19 pandemic, vaccine seems to be the only ray of hope as no specific treatment is available. The scientific fraternity started working on the vaccine and the whole world was eagerly waiting for the vaccine. Now that the vaccine is ready, we are here to discuss what are the perceptions regarding acceptance and expectations from the vaccine among its potential recipients.

It is expected that you feel free to express your opinion. All the information will be kept confidential and anything you say will be not linked to your names and it will not be disclosed to anyone, so feel free to give your comments and your detailed opinion rather than saying just yes or no.

**Opening /Ice breaking question:**

1. What is your name? (In consent form)
2. What is your age?
3. What is your occupation?
4. Have you or any of your family members have been infected with Covid-19?

**Specific questions:**

1. What is your opinion on getting vaccinated against COVID-19 in the current scenario?
   - - - 1. What are the reasons for your answer (Refusal/ Willingness/ Hesitancy/ Eagerness)?
         2. What is your opinion on the concern surrounding the side effects related to COVID-19 vaccine?
         3. What is your opinion on any misinformation affecting the acceptance of Covid-19 vaccine among the community?
2. What are your expectations from the vaccine? (take cues from the answers above)
3. What do you think the government should do to meet your expectations?
   - - - 1. What do you think, when you will be confident/willing to get vaccinated? (What changes can be made in the process?)
4. What is your opinion on compliance to COVID-appropriate behaviour post-vaccination among the community?
5. What is your opinion on the role of COVID-19 vaccine in ending the pandemic?

**Probing of question if needed.**

Can you elaborate that… please explain… anything else…?

**Exit question:**

**We are about to closed interview …**

Is there anything more you want to add that you were not able to say regarding acceptance and expectations of COVID-19 vaccine in this interview?

**De-briefing:**

During this interview, a few aspects on acceptance/ refusal/ hesitancy that you mentioned were ………… and some of your expectations were………………

**I thank you for participating actively in the interview.**

Can I call you for knowing any detail regarding this if required?

**Meanwhile facilitator observer and recorder check note, and audio recording.**

Same day, possible codes will be enlisted as far as possible.
