# Supplementary material for: Acceptance and expectations of healthcare workers and community during the COVID-19 vaccine rollout in Bhavnagar city, western India: a qualitative exploration
Source: BMC Health Serv Res. 2024 Mar 27;24:386. doi: 10.1186/s12913-024-10885-5 (PMC10976747; doi:10.1186/s12913-024-10885-5)
Supplement: Supplementary file 2 — Supplementary Material 2. [file 12913_2024_10885_MOESM2_ESM.docx]

# Healthcare workers

## **Identifier:** **Healthcare worker 01**

**25 years old, Male, Medical officer, SG**

1. What is your opinion on getting vaccinated against COVID-19 in the current scenario?

A. Vaccine for COVID-19 is going to be a blessing for our country.

We have been fighting against COVID-19 since long but now there is a ray of hope in the form of vaccine. There are so many personal opinions regarding vaccine but being a health care worker, I would like to inform that vaccine is a good thing and everyone must take the vaccine.

(a) What are the reasons for your answer (Refusal/ Willingness/ Hesitancy/ Eagerness)?

A. Right now, various myths are spreading regarding the site and side effects of the vaccine, we should not politicize the issue instead we should discuss more that this vaccine is prepared by credible institutes like ICMR, Serum Institute of India, Bharat Biotech and not prepared by some political party. So, by not making vaccine a political issue, we should trust this vaccine and take this vaccine, as we have done with so many other vaccines prepared by scientists, if side effect is seen in one person from lakhs of people, we should not tale it the wrong way and focus on the point that how many people have been benefited from it and all should get vaccinated.

(b) What is your opinion on the concern surrounding the side effects related to COVID-19 vaccine?

A. Every person has their own health status and according to that side effects may be seen in any vaccine; the patient should inform about their health condition and before taking the vaccine. If side effect occurs then is it from the vaccine or due to the health status of the patient that should be made clear and same should be shown via media. Instead of making issue of side effect from vaccine it should be made clear that the effect was due to patient’s health condition, government is well prepared and to address side effects we have prepared a AEFI corner and M D physicians are also available. We all are alert for any AEFI and together we have prepared health teams. We have to convey message that we have AEFI corner, and we are prepared to fight any emergencies and we are ready for it. So, if there is fear in minds of people that what will happen if side effects of vaccine occurs, we are going to provide phone numbers of medical officers and staff to all getting vaccinated and, in any problems, related to side effects they should contact these phone numbers freely and their fears will be addressed. So maximum number of people will take vaccine.

(c) What is your opinion on any misinformation affecting the acceptance of Covid-19 vaccine among the health care workers?

A. The message which is sent to us from above, we are unable to pass on this message to the ground level and because of this there are many misinformation in the staff and if such misinformation is present in the health staff then such misinformation will surely be present in the common public. Much IEC is needed. Much IEC is being done about the vaccine same amount of IEC should be done in people and firstly IEC should be done in health staff that this is the vaccine, these are the side effects of the vaccine and we are prepared fully. If all this information is completely made available to them, then this misinformation would no longer be there.

Even after getting vaccinated we should be responsible. It is not that we are completely safe after taking single dose of vaccine and many in our staff could have this misinformation and every one should understand that only after taking two doses, we can be safe. Even after taking vaccine it is not that we won’t get infected with corona virus, it might lower the severity of the disease if we get infected post vaccination and death might be prevented. We should not be care free that we have taken both the doses of vaccine so we can’t get corona and we have to take care of ourselves even after getting vaccinate.

2. What are your expectations from the vaccine? (take cues from the answers above)

A. Government should make this vaccine available to each and every citizen of India, as soon as possible.

3. What do you think the government should do to meet your expectations?

A. Government has provided target of 100 beneficiaries in one day after much thinking, this should be conveyed down to all the staff members. Beneficiary will get msg at interval of 5 minutes and they will come accordingly. Staff would have to work full day from 8 to 8 and they should be prepared for this and if we work for this much time per day 100 target can be achieved.

(a) What do you think, when you will be confident/willing to get vaccinated? (What changes can be made in the process?)

A. On first basis.

No, there are no suggestions for process of vaccination. Only that the thoughts at upper level should reach to the ground level. How it will be managed at the lower level. Dry run is ongoing but instead of 25 beneficiary it should be done for 100, if it is not done, we won’t know how to do proper time management is done and proper management should be done as 100 cannot be done in a day.

4. What is your opinion on compliance to COVID-appropriate behaviour post-vaccination among us?

A. All of us have taken precautions for this last 8 months and we should take precautions after taking vaccine and we will. Message will be spread about SMS (sanitizer, mask, social distancing in local public too and will have to continue following these precautions everywhere, be it mamta session or another place. whenever a patient comes, we have to provide them is information.

5. What is your opinion on the role of COVID-19 vaccine in ending the pandemic?

A. 100% this vaccine will end this pandemic

6. Is there anything more you want to add that you were not able to say regarding acceptance and expectations of COVID-19 vaccine in this interview?

A. At present, all these things being said by public and all their doubts will be removed by the time all HCWs second FLW and then third stage will be vaccinated

The first two stage are health care workers and front-line workers and side effects won’t happen in them so third stage is going to be easy for us.

## **Identifier: Healthcare worker 02**

**Female, Medical officer, SR**

1. What is your opinion on getting vaccinated against COVID-19 in the current scenario?

A. We have just completed dry run for vaccination. Many people asked us questions and were eager to know, when will the vaccine be available, how will we give it, how much dose will be given and main question of many people was what will be the side effects? Many from our staff are refusing to take vaccine, as there is no guarantee about the side effects and so should they take it or not. There is confusion in our staff about the vaccine and have no idea about how much percent it would be implemented.

B. Vaccine is being launched on a grand scale and government might be 100% sure from their side and so they have launched this vaccine, so one should take this vaccine.

(a) What are the reasons for your answer (Willingness)?

A. Basically, the vaccine has two doses. I myself have searched scientifically and came to know that after getting second dose antibodies are developed after some time and because of this it might be helpful in fighting against COVID-19.

(b) What is your opinion on the concern surrounding the side effects related to COVID-19 vaccine?

A. Main, some death has occurred in foreign country, as this bad news is now amidst people so it is necessary to remove this fear from the people.

(c) What is your opinion on any misinformation affecting the acceptance of Covid-19 vaccine among the healthcare workers?

A. health care workers are confused what is the exact true information and how much percent the vaccine is effective because of the spread of bad news via newspaper and whatsapp in mobile

news in foreign country that someone has suffered allergy, someone has died, one nurse fainted immediately after vaccination. People hold on to this type of 2 to 4 news.

2. What are your expectations from the vaccine?

This vaccine should reach to all the citizens. As there are two doses, planning is to be done on a large scale and proper planning should be done so that people do not get disturbed.

3. What do you think the government should do to meet your expectations?

A. Government has already planned of giving vaccine to 100 people at one booth but to cover all 100 people as per proper protocol is a little difficult.

(a) What do you think, when you will be confident/willing to get vaccinated?

A. As soon as the vaccine is launched.

(b) (What changes can be made in the process?)

A. Main is about entering data into portal as sometimes network issue occurs or servers might be down. At present we don’t know how much easy it would be, we will come to know only after its launch.

4. What is your opinion on compliance to COVID-appropriate behaviour post-vaccination among us?

A. Would necessarily have to follow COVID-appropriate behaviour.

5. What is your opinion on the role of COVID-19 vaccine in ending the pandemic?

A. We can be hopeful that this vaccine will end this pandemic.

6. Is there anything more you want to add that you were not able to say regarding acceptance and expectations of COVID-19 vaccine in this interview?

A. Mainly people have fear in their minds about what would be the side effects of this vaccine and how will it be launched, what is the procedure etc.

So, more and more publicity should be done that this vaccine is safe and it would be good if this message is being spread.

## **Identifier: Healthcare worker 03**

**24 years old, Male, GNM, PB**

1. What is your opinion on getting vaccinated against COVID-19 in the current scenario?

According to me , the vaccine which is arriving and is going to start from 16 th January in that we have included people over 50 years they have to compulsory take vaccine and also those who are below 50 and have co morbidities. But all people in community wont be ready to take vaccine as they might be believing in some superstitions about content of vaccine and some believe that we do not want to take vaccine as there is no such thing as corona. And if after taking vaccine if death or other side effects occur who will be responsible for our family.

What are opinions about vaccine in health staff?

Vaccine has come and been told that it is compulsory to take vaccine for health staff , but all are not ready to take vaccine. I myself am not ready to take vaccine because I have been working in hospital and field and have already come in contact with positive corona patients and i am well. So what will be the significane of vaccine for me.

(a) What are the reasons for your answer (Refusal/ Willingness/ Hesitancy/ Eagerness)?

A. There might be other more side effects and so there is fear about that. it is not that if I don’t take others wont take but I don’t want to take vaccine.

(b) What is your opinion on the concern surrounding the side effects related to COVID-19 vaccine?

Vaccine is arriving and vaccination is starting from 16th January. We cannot say with 100% surety that there wont be any side effects. Side effect can occur 1 in 1000 or so, major or minor. But we can’t say anything about major or minor side effect at present.

(c) What is your opinion on any misinformation affecting the acceptance of Covid-19 vaccine among the healthcare workers?

No idea about that.

2. What are your expectations from the vaccine?

What every company launches vaccine there should be hundred percent result of the vaccine. And cases of corona decreases, it has decreased now but the symptoms too decreases and the people in age gropu above 50 years or with co morbidities such as hypertension, diabetes comes in vulnerable group. So vaccinating them can lead to their early recovery.

3. What do you think the government should do to meet your expectations?

Maximum population should be covered and all should get vaccine. Govt is spending right now for health of all and if every one takes vaccine it will be better.

(a) What do you think, when you will be confident/willing to get vaccinated?

A. Am not saying that I will take compulsory. My mind says that I have worked till now and I have not showed any symptoms till now. I too have been tested by rtpcr and rapid and haven’t seen any symptoms in me. But there is fear that if there are some side effects major or minor. So, I don’t believe that I can take vaccine.

(b) (What changes can be made in the process?)

A. The planning done at present is okay. Various community places are decided for session site. And at present whatever the population is to be covered by that site, the list is prepared by online entry of people over 50 years and people having comorbidities all should get proper service and all get vaccinated and we get good results.

4. What is your opinion on compliance to COVID-appropriate behaviour post-vaccination among us?

A. It is most important. Corona vaccine has arrived it does not mean that we are free from corona. We have habit of wearing masks, frequent hand washing, 2 feet social distancing and should continue doing it. if we follow this then people don’t come in direct contact with each other as we can not say about sign and symptoms of corona, also other communicable disease can’t occur.

Q. as you said earlier you are not willing to take vaccine, when will you be ready to take vaccine (after what changes?)

A. vaccination has just started, after some vaccination occurs then we could be aware about the side effects and its effectiveness and based on that we can get somewhat idea that it is working properly or not.

5. What is your opinion on the role of COVID-19 vaccine in ending the pandemic?

A. Corona was there, at present time of corona is up. If we see in Bhavnagar in the community there is noting like corona. People do not believe that there is anything like corona and they tell health staff that you come here without any reason. We tell them to wear mask, wash hands as 8 to10 people are living in the same house but they do not follow any of this instruction. We also guide people who come here or the people nearby but they say that now there is nothing like corona and corona is gone.

6. Is there anything more you want to add that you were not able to say regarding acceptance and expectations of COVID-19 vaccine in this interview?

A. No, nothing else.

## **Identifier: Healthcare worker 04**

**28 years old, Female, ANM, RS**

1. What is your opinion on getting vaccinated against COVID-19 in the current scenario?

A. The vaccine introduced by the government is for our benefit only, proper research has been done and only then it is made available to the public. So, it is our responsibility to give response.

Q. what is your personal opinion on taking the vaccine?

We are health care workers and it is our responsibility to support the vaccine. Even if we keep the responsibilities aside and think like a common man, the vaccine is for our benefit only government has no profit from this. Vaccine is for us and if we don’t support the government won’t be able to do anything in this. If we want to live a normal life then we would have to take it. This is pandemic and it is for every one not for a single being. If it was for 1 percent then it would be okay but it is at India level and so we have to support

(a) What are the reasons for your answer (Willingness)?

For my self not for any other person. If I want to life a good life then we have to support this. In the lock down we were not able to go out from our house, were not able to mee our family members, we would have to stay away from our family so that they do not get infected, if we don’t want this to continue for long time then its is essential to get vaccinated.

(b) What is your opinion on the concern surrounding the side effects related to COVID-19 vaccine?

In any vaccine or even in any medicine there will be side effects in some people and that is a normal thing. If we talk about allergy then it is dependent on, to whom and in how many percent side effects occurs as ever person has different immunity level. I have heard that in ten lakh people side effect will occur in ten people and we don’t know if we are in those ten people. we will come to know only when we take vaccine and even if we are in those 10 people our team will be ready and we will get immediate service and we will get better. If vaccine is effective then we will be able to live good life in future and if present situation continues then there are chances of our death in short term.

(c) What is your opinion on any misinformation affecting the acceptance of Covid-19 vaccine among the healthcare workers?

A. If we talk about hoaxes, they are going to spread in any situation. It is not that it spreads only in medical field it spreads in any field. The people spreading hoaxes will continue to do so and they wont listen to us if we tell them not to do so. But it is on us whether to listen to that or not. Someone has told us some thing, we don’t know wheter it is true or false or neither we have experienced it. If we have experienced it then we can tell anybody that we have done this and all this thing occurs. I myself have not taken the vaccine and before taking the vaccine if I say that this vaccine is not good and don’t take the vaccine, there are five members in my family if I don’t take the vaccine other five family members are not going to take vaccine, and this chain will only get bigger, their friend circle and ten their friend circle, so we should not spread negative things.

I believe that vaccine is for good purpose and we should take it. We should convince others too in this way.

Which hoaxes have you heard?

I have heard that there are chances of death after taking this vaccine and the vaccine is not successful and even then, it is being launched in India.

But it is not like that, we have all this big research labs and they won’t make this vaccine available to public without proper trials and govt would never approve such thing so we have to think with our mind that nobody exposes us directly like this. And if step by step things are done, we will get it in last stage we can’t think anything more about this.

2. What are your expectations from the vaccine?

Hopefully I thing only one thing that the vaccine successful. And till now we have heard only hoaxes that the vaccine is successful and it is in the last trial. it can be a hoax or it can be true. We don’t want to oppose or anything we would even take the vaccine but would hope that it is successful and we get that vaccine.

3. What do you think the government should do to meet your expectations?

Government should take actions against people spreading hoaxes. There are some person whom people trust and they believe that they only speak the truth such people should go in between the people and come in direct contact with people and explain them. If ten people are unwilling at least five of them would become willing if these person talks with them.

As you said, what other things can be don’t to spread awareness?

A. Through news, Whatsapp and from young generation to old everyone uses internet so by making various channels on the internet, proper guidance and aid be sent to such channels then maybe there are chances that people will get convinced.

(a) What do you think, when you will be confident/willing to get vaccinated?

A. Anytime. When ever the vaccine arrives and we are told to take the vaccine at a time we are ready to get vaccinated

(What changes can be made in the process?)

No. already govt of Gujarat has taken a good decision that first vaccine will be given to health care workers and frontline worker and after that it will be given to people above 50 years of age, we have prepared the list and it will be given accordingly and it is wright decision and we support it. if we have taken the vaccine, we will be able to tell ten other people that I myself have taken this vaccine and nothing has happened to me and so nothing will happen to you too. We can call it live demo and we as healthcare workers will be standing there a s live demo.

4. What is your opinion on compliance to COVID-appropriate behaviour post-vaccination among us?

A. Even after taking the vaccine we have to follow all rules as we have been doing and we don’t have to bring any changes in that , wearing mask, washing hands repeatedly, staying away from crowded places and even if we go to crowded places we have to take precautions like social distancing, all this things are to be followed until we get relaxations for our wellbeing and we should take it as our responsibility.

5. What is your opinion on the role of COVID-19 vaccine in ending the pandemic?

Vaccine has come as a ray of hope and hope that it is successful and the cases which are rising now from medium to higher side that can be brought under control then it is better. We can be COVID-19 free because of this vaccination.

6. Is there anything more you want to add that you were not able to say regarding acceptance and expectations of COVID-19 vaccine in this interview?

no nothing about that. i have accepted at present but we have to people accept it and we would have to explain then in their language, there will be many controversies and frictions with people and if side-effects occur in someone then we are going to be the debate too. when people come to us for vaccination we would have to convince them and set some example so that their self-confidence also increases and so after that there are chances that acceptance increases.

## **Identifier: Healthcare worker 05**

**31 years old, Female, ANM, MB**

1. What is your opinion on getting vaccinated against COVID-19 in the current scenario?

Vaccine should be taken. Vaccine is good.

What are your personal views?

It is good if we take this vaccine as cases of corona are rising and many people have died too. we get immunity from vaccine to it is necessary to take vaccine

(a) What are the reasons for your answer (Refusal/ Willingness/ Hesitancy/ Eagerness)?

We have to work in field and we come in regular contact with patients and we cannot wear mask and all things all the time and we come in contact anyhow. So it is necessary to take vaccine.

(b) What is your opinion on the concern surrounding the side effects related to COVID-19 vaccine?

Side effects occurs in some people with comorbidities.

Have you heard anything about side effects?

(c) What is your opinion on any misinformation affecting the acceptance of Covid-19 vaccine among the healthcare workers?

Hoaxes are spread more dur to media as they show it out of proportionate ans such many things are occurring right now. Many people are refusing to take vaccine and say that we don’t want to take the vaccine and many people are coming forward and saying that they want to take the vaccine.

What hoaxes have you heard?

Recently I had heard that vaccine was given to a person and he died and also people tell that if something happens to us then who will take responsibility. In case we give vaccine to someone and something happens to them they are going to tell us as who will take the responsibility as we have given the vaccine. Many people say that if you take responsibility then we will take the vaccine. There are many people like that too.

Would you say more about this?

There are many hoaxes right now people are also saying that there is no such thing as corona these people decide by themselves positive or negative. Even we don’t have corona these people will tell you are infected with corona and would throw us.

2. What are your expectations from the vaccine?

No side effects occur to people and all live in a good way and we could win against corona.

3. What do you think the government should do to meet your expectations?

Should see that no side effects occur from the vaccine.

What steps can be taken?

(a) What do you think, when you will be confident/willing to get vaccinated?

We are ready when the vaccine comes, we are going to take it.

(b) (What changes can be made in the process?)

No. we have conducted dry run and everything is alright.

4. What is your opinion on compliance to COVID-appropriate behaviour post-vaccination among us?

We should definitely follow all these steps for safety as if someone has it and we are infected with it.

5. What is your opinion on the role of COVID-19 vaccine in ending the pandemic?

No nothing else.

6. Is there anything more you want to add that you were not able to say regarding acceptance and expectations of COVID-19 vaccine in this interview?

Care should be taken that correct information reaches public and not the wrong information. And if something occurs to even a person, death or any other big issue occurs then no one will take the vaccine. So, we should take care of this.

As you said correct info should reach to people and not wrong info what can be done for this?

All that depends on vaccine and what is the effect of vaccine on patient.

That can reach people via media and health staff.

Any suggestions from you side?

Health staff and ASHA workers are from village and so people believe them more and so if these people spread information, then it is better.

## **Identifier: Healthcare worker 06**

**26 years old, Male, Resident Doctor, JK**

1. What is your opinion on getting vaccinated against COVID-19 in the current scenario?

People have been waiting since long for vaccine to be found so that we can get rid of this pandemic. At present we are waiting for distribution of this vaccine and the scale of pandemic decreases.

What is your personal view about vaccination?

For covid 19 we give so many drugs and some big injections like tocilizumab etc but even then, some people die. If we can get rid of its root cause the virus, i.e. if we are able to develop immunity in our body against the virus, vaccine will be very effective.

We can stop the disease before it occurs. After some age and people with co morbidities such as diabetes, hyper tension etc if they get infected with covid then it is very difficult for them to recover from it.

(a) What are the reasons for your answer (Willingness)?

I expect that health care workers gets vaccine early; doctors nurses and other workers of hospital gets the vaccine first then general population also gets the vaccine so that this disease can be prevented and we can decrease mortality and morbidity.

What is your opinion on vaccine?

Many people are saying that there are many side effects, but until we experience such things, we won’t be able to know.

(b) What is your opinion on the concern surrounding the side effects related to COVID-19 vaccine?

A. If we get the vaccine early then we will know about the side effects i.e. what and how many side effects occurs. We will know more only after some experience.

(c) What is your opinion on any misinformation affecting the acceptance of Covid-19 vaccine among the healthcare workers?

A. At present many hoaxes are spreading that vaccine for covid has so many side effects some people say that in long time it damages kidney, it also affects CNS, but still vaccine has not been given and so we cannot know till then.

Have you heard any such misinformation?

I have heard that in long time it damages kidney, it also affects CNS but it has not been proven scientifically.

2. What are your expectations from the vaccine?

I expect that the man power being used in this pandemic can be decreased and the cases can be decreased. The cases are decreasing slowly but because of vaccine another wave of COVID-19 cases can be prevented.

Can you say more about man power being used?

Cases of COVID-19 rises so much that we cannot give our output in other disease, as equal man power is required in that too, doctors nursing staff etc. Plus, we have to work wearing PPE kits and it is very difficult for everyone. Wearing PPE kit is a challenge.

3. What do you think the government should do to meet your expectations?

Government should enforce rules about masks and if another wave occurs then government should impose lockdown.

About vaccine?

About vaccination, if the vaccine has arrived then start vaccination as soon as possible and give vaccine to people who requires it primarily like doctors and nursing staff.

(a) What do you think, when you will be confident/willing to get vaccinated?

I am ready whenever the vaccine comes, I will take it.

(What changes can be made in the process?)

When the vaccine is given it should be noted by number or something and it should be given at a specific site ay CHC or tertiary care centre and not everywhere so that misuse of vaccine does not occurs.

4. What is your opinion on compliance to COVID-appropriate behaviour post-vaccination among us?

Even after vaccination we would have to follow rules as we don’t know the efficacy of vaccine and if the vaccine is 100% effective and COVID-19 19 is preventable or not. We would have to continue wearing masks and all things.

5. What is your opinion on the role of COVID-19 vaccine in ending the pandemic?

Effect can be there. It seems like it has 60 to 70 percent effective, if heard immunity is be developed then there are more chances of preventing covid-19.

6. Is there anything more you want to add that you were not able to say regarding acceptance and expectations of COVID-19 vaccine in this interview?

Firstly, vaccine should be free of cost. All people should get it but stepwise. And it should not happen that people at village level or living in difficult to reach places does not get vaccine, as all people can get infected with covid. We should spread awareness about the side effects of vaccine in the patients too that this king of side effects can occur.

What can be done for spread of awareness?

For awareness all advertisement can be done there are many ways, radio ,tv, we can give ads on public channels for e.g. we give ads for polio in tv that there is a polio day and vaccine for polio will be given at these places.

## **Identifier: Healthcare worker 07**

**29 years old, Female, Medical officer, SP**

1. What is your opinion on getting vaccinated against COVID-19 in the current scenario?

At present there are two sides to the vaccine on one side there is fear in the public about the side effects of the vaccine and on other sid ewe were waitng for the vaccine that when it is available and when it brings an end to this corona. If we take vaccine antibodie are generated in our body , as body reacts to vaccine and the chances of getting infected decreases

Can you say more about the vaccine?

Some people have low immunity so for increasing immunity it is very important to get vaccinated

(a) What are the reasons for your answer (Refusal/ Willingness/ Hesitancy/ Eagerness)?

(b) What is your opinion on the concern surrounding the side effects related to COVID-19 vaccine?

And about side effects it is not necessary that side effects have occurred due to vaccine many a times, due to personal phobia there are effects on the body. Many a times even if needle has not even touched them, they feel like they have been pricked and same occurs in vaccine, that side effects has occurred in one person then that will happen to me too. and it is not that side effects has occurred because of vaccine. People should understand this and should think deeply about this.

(c) What is your opinion on any misinformation affecting the acceptance of Covid-19 vaccine among the healthcare workers?

I will only say this that awareness should be brought in people that side effects do not occur after taking vaccine. When health care workers will come forward and get vaccinated then common public will also get in line to take the vaccine. To remove this fear vaccination is necessary.

2. What are your expectations from the vaccine?

Because of vaccine against corona immunity in people will rise and we would be able to defeat corona

3. What do you think the government should do to meet your expectations?

Government should first spread awareness using banners or by using social media and by front line workers or through politicians / social workers and people get this message and then we can get fully successful in this vaccination programme.

Any other suggestions for bringing awareness?

As I have already said for awareness use of social media should be done and that will bring awareness in people. It is necessary for government to totally stop negative news and hoaxes as this can lead to fear in public and people may refuse to take vaccine.

Have you heard any such hoaxes?

It is being said that corona causes other diseases too but it has nothing to do with corona. Same thing happens in vaccine, if some disease is going to occur and at same time person gets vaccinated then people can get misguided that the disease has happened due to vaccination but in reality, it is not so. Doctors should make people aware about this.

(a) What do you think, when you will be confident/willing to get vaccinated?

A. When vaccination programme is implemented by the government, we are ready to get vaccinated.

(b) (What changes can be made in the process?)

A. The process right now is well planned that at a time only one person gets vaccinated and that person is kept in observation. It is necessary that this is implemented by all.

4. What is your opinion on compliance to COVID-appropriate behaviour post-vaccination among us?

A. After getting vaccine it is important to follow the guidelines because even if you are immunised other people can be infected such as you family members and others so it is necessary for the shake of others that we wear masks, use sanitizers, wash hands frequently.

5. What is your opinion on the role of COVID-19 vaccine in ending the pandemic?

A. Person can get complete protection by corona vaccine so it is necessary to get vaccinated.

6. Is there anything more you want to add that you were not able to say regarding acceptance and expectations of COVID-19 vaccine in this interview?

A. There is one thing that as the vaccination progresses the fear in minds of people will decrease and people will come forward to get vaccinated. At present there is too much fear (હાઉ), when corona first stared and people were not coming out of house and were feeling insecure but as time passed people stated accepting it and fear and thought about corona started decreasing in the minds of people. Same condition will happen in this. First some people will get vaccinated will observe them and then people will come forward themselves for vaccination.

what can be done to remove fears from the minds of people?

The people who have got vaccinated and has no side effects, with the help if interview or media it can be spread in people. We are promoting people to take vaccine in the same way this can be promoted. If Amitabh bacchan or some cricketers take the vaccine and that gets viral in social media their fans will follow them and surely get vaccinated.

## **Identifier: Healthcare worker 08**

**34 years old, Male, Medical Faculty, DC**

1. What is your opinion on getting vaccinated against COVID-19 in the current scenario?

At present, for vaccination government is planning regarding it and as per current condition vaccine is the only condition which can control the pandemic along with limited cases. For government and vaccination related, according to my perception and personal opinion I am not willing to take it and as I am a medical person I will not suggest regarding vaccination to any of my family members.

Second thing is that if I am not that confident in this thing that I should take or not uptill than as a medical faculty it is a government program so according to ethics I will suggest other to get vaccinated. Personally as a citizen of India I am not preferring to take the medicine. Now the question would be why do I not want to take vaccine? To not to take vaccine I would prefer the question that why should i take vaccine as me in person? Than I should take for myself this disease is totally new. For the first time I am having chance to get in high risk in that condition I am having zero immunity, in that case I will prefer the vaccine. Since the time I am doing the job as a medical faculty/doctor, in this COVID era since 1 year, according to my perception I have already been in contact with COVID and I would already have antibody which will work as vaccine for me. So there is no need of taking vaccine for me only. Second thought would be that why would I not suggest regarding it to others? For that I am not having confidence on vaccine. For not having confidence I can say that government has kept many details confidential till date. There is not any clarity for this like as a citizen/ medical practitioner I do not know about side effects of vaccine. Till yesterday night I didn’t know regarding route and dose of vaccine or supply of vaccine so off sudden anyone would say that this vaccine is there and you should take which is not acceptable for me. So I am not saying that I would never take this vaccine. I would take the vaccine when I would have confidence on it because according to me confidentiality present in trials like how many number of people they have studied on or what is reason or what are side effects or 1% serious side effects if present than why is present? This people should say that if side effects not present than why are not saying that not present. This is like a story that elephant was killed or aswathama which we do not know. So in whatever amount we remain positive but in this pandemic at present this risk should not taken according to me. So at home I would say that at present it is not there, I am not having enough evidence that we should take vaccine. Another thing is there is lack of IC is present regarding the vaccine.

As you stated that there is lack of IC, enough information regarding IC is not available than according to your opinion for getting enough IC, what should we do or what can be done for the same so that information can be passed to last person?

Basically whichever vaccination programs are conducted like in recent when MR vaccine was there, Work for MR vaccine was very big because for individuals less than 15 years vaccine had to be given for that programs were done in schools and less than 15 years means around 21% population is there, in Gujarat itself to around 2.5 crore individuals vaccine had to be given and at that time we had fear that all will take or not and at that government had taken steps at Gandhinagar like stake holder which means Commissionerate or CDHO or under them officers is considered to be stake holder. So they were called to Gandhinagar and taken into confident. Not even them but media was also called and taken under confident. After they were made confident like CDHO than after that over here like employees under CDHO like Medical officer from CHC and PHC were called and made them confident, over here local media was made confident, over here local corporaters were made confident but after that people under them like social worker explained to people that vaccine is safe, Medical officer also explained to people that vaccine is safe and you should take it to people, people working in ANM like asha worker was also made confident by MO. Because when MO will make confident to Asha than they will explain to local people regarding vaccine than this was the challenge two years ago. This talk is regarding around 2018 that this process was done than question is also arising that at present many virtual meetings were conducted. We can understand that social distancing is necessary so big virtual conferences were conducted. So why these big virtual meetings have not been conducted which is the big question arising in myself.

What is your personal opinion on getting vaccinated against COVID-19?

According to my opinion uptill when all will be not made confident, I am having confusion as all would be having. As a medical officer/ doctor we are also stake holder if we are having questions than we can’t give answers to layman people so that’s it.

What is your opinion on the concern surrounding the side effects related to COVID-19 vaccine?

For side effects we already know that at injection site there is redness and swelling present, small or big sterile abscess can occur, other nausea or vomiting can occur, fever can occur. But as a doctor I have not read about it anywhere, actually for any drug present there is a separate column present for side effects like common side effects or rare side effects. Like fever is there than how much common it is like 90%,95% or 99%, vomiting is there than how much it is common, there is no such type of data even as a doctor I don’t know. I don’t know regarding it so I cannot say anything.

2. What is your opinion on any misinformation affecting the acceptance of Covid-19 vaccine among the community? What can be done to minimize such rumours?

Actually I have already given answer like rumours are present like delusion rumour. Actually, there are two sides like if a talk is negative than it becomes a rumour and if it is positive than it becomes IC. If Ic is done than automatic rumour will be neutralised and will go towards positive side so as I said you should take all the people into confidence.

3. What are your expectations from the vaccine? (take cues from the answers above)

Expectations from vaccine can be taken as actually it is a kind of flu vaccine and we have already seen flu vaccine for swine flu like there is a limit for 2-3 months of it that first expectation from vaccine should be that 3 months is not enough. For example you are taking vaccine now, I roughly know about it, second dose will be given after 1 week, effect of vaccine would come into action after 10-15 days and will be effective for 3 months. If we include it than for around 2.5 months vaccine will be effective than after that again. we have to again take vaccine uptill how much time it will be done. How much % it is effective that I don’t know but it would be more, our government and scientist, even laboratories are working on it than we should keep expectations that vaccine should be long term effective and maximum efficacy should be there that after taking vaccine few people would develop disease. That’s it.

What do you think the government should do to meet your expectations?

Government could not do anything more in it because talk is that 3 month efficacy is only there so I can’t suggest that government can do anything in it. Government can in a proper way through a proper channel develop confidence in people and IC distribution, though less number of it is present but people can take vaccine.

What do you think, when you will be willing to get vaccinated?

Actually this is lack of data and IC, when I would gain knowledge that it is safe and effective and efficient on that day I would get vaccinated.

What changes can be made in the vaccination process? Any suggestions?

I already said that in a proper way it is done than it would be more effective.

4. What is your opinion on compliance to COVID-appropriate behaviour post-vaccination among us?

Covid-19 is a re-emerging disease which came suddenly so we don’t know enough about it, so vaccine for it would not be more efficient like for MMR second dose is giving 99% efficacy, first dose is giving 93% efficacy than this vaccine if made one of the best than would not be more than 70-80%, it means out of 100 people 70 would not develop and 30 will develop but in 30 people you have to decide that he/she has developed or not, if not developed than COVID appropriate behaviour should be kept.

5. What is your opinion on the role of COVID-19 vaccine in ending the pandemic?

Actually, for vaccine, I would remember about swine flu in which vaccine was of this type in which limit of 3 months was there. At that time, I had also done work at OPD and ICU setup. At that time although doing work I had not taken vaccine of it, it doesn’t mean that I am having problem with COVID vaccine. At that time I had not taken in the same way we have been doing a lot of work and antibody would already be present in body than why it is necessary. For the vaccine if it would be long term effective and efficacy would be there than it will play a role other than that vaccine would not have that much role, other than that there is a chance that we can do herd immunity, another Covid behaviour. Overall, as a doctor we can suggest that healthy behaviour for Covid than time would come when COVID cases would be decreased and we would be that much immune that Covid would not affect us or we would not get symptoms of it.

6. Is there anything more you want to add that you were not able to say regarding acceptance and expectations of COVID-19 vaccine in this interview?

No, we have already done it.

Thank you for taking participation in this activity. Thank you, sir.

Thank you.

## **Identifier:** **Healthcare worker 09**

**32 years old, Male, Medical Officer, CS**

1. What is your opinion on vaccination against COVID-19 in the current scenario?

Vaccination is very good thought and it has to be done. In this pandemic, when there is no specific Medicine, Vaccination is the only salvage. (Vaccination E j Bachav che!) That's why vaccination is good and it has to be done.

What is your opinion on getting vaccinated against COVID-19 in the current scenario?

In my opinion, 100 % we should take it.

What are the reasons for your acceptance of Vaccine?

As I have told you earlier, We have no specific Medicine for COVID-19 infection, Vaccination is the only salvage. (Vaccination E j Bachav che!) It's the main reason for same.

What is your opinion on the concern surrounding the side effects related to COVID-19 Vaccine?

In any Vaccine, minor side effects are there. Minor side effects are being accepted, no such big issue with it. And trials have already been done. As per knowledge, all 3 phase of trial are accomplished. No major side effects was found in clinical trials. So there is no such issue.

What's us your opinion on any misinformation affecting the acceptance of COVID-19 Vaccine in health care workers?

Yes, few are misleaded. Especially health care workers of lower classes are misleaded. But it can be managed with persuasion. No issue with it. If we can proper guide them and explain well that trials have been done and no Major side effects were observed, than it is possible.

Have you heard such misinformation/Rumours about COVID-19 Vaccine?

Mainly Rumours are heard from newspapers as well as other people. Like due to COVID-19 Vaccine, COVID-19 infection is transmitted. Even it was heard that deaths are occurring due to vaccine. After publishing the news of Britain and other places where Vaccination has already started and Side effects were noticed, negative impression was observed more as per my opinion.

2. What are your expectations from the vaccine?

We can have major Result (impact), if effective result of Vaccine are noticed in atleast people with Comorbidity and people with age more than 50 years. It will play a major role in breaking the chain of COVID-19 infection.

3. What do you thun the government should do to meet your expectations?

As I said, maximum vaccination coverage should be done in atleast people with Comorbidity and people with age more than 50 years.

What do you think, when you will be willing to get vaccinated?

I think, on the day of inauguration of COVID-19 Vaccination program on 16th Jan, health care workers are about to enrolled for Vaccination. I am planning to get Vaccine on the same day.

What changes can be made in the vaccination process? Any suggestions?

I have seen the vaccination planning and we have organised the dry run too, where I was one of the participants. So no such specific suggestion from my side. Almost everything is fine.

What can be done to increase Vaccination coverage in people with Comorbidity and people with age more than 50 years?

Survey for this Population is already done and we already ahve the list of such beneficiaries. So once we have planned the session and Beneficiaries are informed about it with message or call, we should take follow up of them. Ex. They have visited the session or not? They are actually vaccinated or not? If not, then why? We should be knowing their reasons for denial. we can call them again for Vaccination after clearing their dilemma/doubts with proper explaintion.

4. What is your opinion in compliance to COVID appropriate behaviour post vaccination in Health care workers?

According to me, Covid appropriate behaviour should be followed even after Vaccination. Safety etiquettes like Mask, 6 feet distance etc should be followed for upcoming one or two years.

5. What is your opinion on the role of COVID-19 Vaccine in ending the pandemic?

Vaccination will play a major role. 100 percentage. As we look into results of trials, it seems we will get complete success in vaccination.

6. Is there anything more you want to add that you were not able to say regarding acceptance and expectations of COVID-19 vaccine in this interview?

I think proper awareness we required is not there in general public. So acceptance level would be very less in them. In my opinion there would only 50% acceptance. The main reason is negative news from newspapers or heard it from different people. This will play a huge role.

What is your opinion on acceptance of vaccination against COVID-19 in health care workers?

In intial time, even health care workers were worried after hearing such negative news. But with the help of their medical knowledge and our proper explaintion, we can persuade them. I think there would not be much issue in health care workers. There may be issue in intial sessions. After that explaintion and witnessing the condition and knowing how's the situation of beneficiaries who have vaccinated in intial phase, immediately all will accept it too.

## **Identifier: Healthcare worker 10**

**30 years old, Female, Medical officer, RJ**

1. What is your opinion on getting vaccinated against COVID-19 in the current scenario?

At present, according to my opinion on 16th date vaccine is going to launch,my personal opinion is that I am going to take vaccine first after that by observing me my staff will take vaccine so first of all vaccine is necessary for prevention and in my family no one has developed disease so for prevention it is utmost important to get them vaccinated.

What are the reasons for your Willingness?

First, we have to constantly work in field, dealing with patient is there. So which patient is positive or negative we do not know.

Second thing is we do Rtpcr or rapid antigen test than 90% patients will come positive for that we already wear PPE kit in which only one mask is present, gloves are also not there so there are more chances to get infection than after taking vaccine we are getting chance to prevent infection than why should we not take benefit of it.?

What is your opinion on the concern surrounding the side effects related to COVID-19 vaccine?

In side effects mainly according to me anxiety. If more anxiety is present than vasovagal shock can be occurred other than that General side effects are present. TT or hepatitis may be occurred at any first time.

2. What's us your opinion on any misinformation affecting the acceptance of COVID-19 Vaccine in health care workers?

Rumours like many health care workers are discussing that after taking this vaccine if we would develop anything than what would happen to our family. We are health worker so first trial on us is wrong thing which should not be done. According to me, we are health worker than first we are at higher risk than our family so we have to take vaccine and I also give advice to them and for that at least for my staff I am trying that my staff should take vaccine, I do counselling of them so they would take vaccine.

As you stated that ‘why should I be first? ‘ which is told by some health workers so what can be reasons for the same?

First any other would get vaccinated than after observing them we will take vaccine this type of thinking is there among some health care workers. They would think that any bad thing does not happen to them. For example on myself first trial is done than any problem occurs than it would only occur to me. After another person gets vaccinated after observing them I will take vaccine this type of false belief is there which we have try to break it. Any vaccine is there than direct trial is not put on us, we are the first step as a corona warrior so government would not keep us in danger by doing direct trial on us so government would had kept trial for it at any other place after that implementation of it would be done on us.

Have you heard such misinformation/Rumours about COVID-19 Vaccine among health workers?

Everyone is in fear that if this vaccine would cause any serious side effect on them than what will they do. Common side effect is ok but if direct serious side effect occurs than we would like to have COVID positive but not any other serious illness. They are in more tension that after taking it they would not develop any other serious effect.

3. What are your expectations from the vaccine?

Atleast, like polio vaccine is given than it is not necessary than we cannot get polio but atleast about 90-95% we have cured it, like that in 90-95% in these cases after taking corona vaccine we can do prevention of disease than it is more good for us that we would have immunity, social distancing, mask along with these vaccine can be considered as a role. After having vaccine it is not that mask and social distancing is not necessary and we have won against corona but atleast after vaccine antibody is developed in body which would play around 70-80% role in prevention in body than in future it is going to help us in any field like for example in mass communication is there than we are having plus point that we are cured by having vaccine and we would develop positivity in ourselves that we have taken vaccine. And due to COVID people is not facing illness on its own but due to fear of Covid he/she gets illness. If common cold is present than in them fear would be present like that positivity can be developed by vaccine in them. Like they would think that they have taken vaccine so it would be common cold and not corona infection or common URTI can be present. Direct fear of it will not develop in them.

What do you think the government should do to meet your expectations?

If After taking vaccine any serious side effect occurs or AEFi occurs than it is necessary that implementation of it should be done immediately. Improvement should be done instantly which is necessary, in which ratio AEFI is present which is necessary.

What do you think, when you will be willing to get vaccinated?

On 16th vaccination is to be done than for that I am already ready for it.

What changes can be made in the vaccination process? Any suggestions?

During vaccination procedure, 10-15 mins are taken for whole vaccination process to be done from waiting to get vaccinated.

Main thing is observation. For that if sufficient observation facility should be made available by government. If observation of 30 mins is kept in a room where only 5 people can be kept than only 5 people would be there in room than after 30 mins another 5 people will be kept. So more time is wasted so according to me if government would have more observation facility than more speed would be present of vaccination.

4. What is your opinion on the role of COVID-19 Vaccine in ending the pandemic?

Hopefully vaccine made by government and scientists would be 60-70% effective but more positivity would be there which is according to my thinking. Atleast through it 40% positivity would be developed in them that they have got vaccinated and fear in

them would get away. As we have seen that individuals of above 50 years with diabetes or any comorbidity they would have first fear that they will not live which would decrease in them.

What is your opinion on acceptance of vaccination against COVID-19 in health care workers?

At least first as a corona warrior government has give us salutation, out of our country what will be cost of it we don’t know but at least to us it is given free of cost than we should take it. And government is not doing trial on us so we should accept it and take the vaccine. As to new borne baby instantly we give BCG to baby in the same way government has given us first priority as a staff member than we have to take the vaccine.

Thank you for taking participation in this activity. Thank you sir.

Thank you.

## **Identifier: Healthcare worker 11**

**26 years old, Female, ANM, PP**

1. What is your opinion on getting vaccinated against COVID-19 in the current scenario?

Public is annoyed in COVID-19 pandemic. Families are also thinking that this vaccine will be helpful as preventive and curative.

Some people are also protesting that this Vaccine is fake. It has side effects. Person dies due to Vaccine. Other diseases may occur with this vaccine. Such misinformation are there. I don't believe that such things are possible.

What is your personal opinion on getting vaccinated against COVID-19?

If government is thinking about public, than this Vaccine must be good. There is no issue in accepting this Vaccine. As a health staff, I want to say that if we are taking this vaccine, than there should be no issue in accepting this vaccine in Community.

What are the reasons for your answer (Refusal/ Willingness/ Hesitancy/ Eagerness)?

What is your opinion on the concern surrounding the side effects related to COVID-19 vaccine?

We are also doing normal vaccination. In that too, few side effects are there. Any external elements is introduced in your body, antibody response will be produced. It shows its effect only if side effects appear. Even with normal injection, swelling is very common. (Normal injection lyo ema bhi dhoklu to thay j che!) Everyone has different reviews like Dhoklu (Swelling) is there, Swelling has occurred, Redness is there.

With his Covid19 Vaccine, If someone has side effects like swelling or redness, treatment will be delivered to them. If someone has phobia to vaccine that if i will take this Vaccine, I will suffer from this issue, convulsion may occur, or giddiness or breathlessness may be here, all of this can be treated with AEFI kit.

2. What is your opinion on any misinformation affecting the acceptance of Covid-19 vaccine among the community? What can be done to minimize such rumors?

First of all as a health care worker, they should not listen to such rumors as they are working in health system. These rumors are all fake.

I have heard that one Health staff who was a volunteer for the Covaxin Vaccine trial is dead due to breathlessness in Bhopal, Madhya Pradesh. They are saying such trail should not be done in Health staff first. After hearing this rumors, our family members called us that if such Vaccine is given to health staff, don't take it. As such news are coming, what if something happen to you after Vaccination? Other rumours is also there like patient goes to coma due to Vaccination. This is also fake. As no one has really seen the actual Vaccine nor anyone have taken it.

Such rumours are baseless.

To minimize such misinformation, we can arrange seminars. But as we have to maintain Social distance so we can't gather people. But as a health care worker, while visiting schools or Aganwadi center, we can give Health education. We can also give advertisement for same in TV, which can be done by Health leaders like Harshvardhan Jadeja sir or locally well-known Medical officer of area. Medical officer of Subhashnagar UHC is very well known face in our area. If she convinces and provides health education to seminars or any small gathering, people will agree to her.

3. What are your expectations from the vaccine? (take cues from the answers above)

It's not like Covid19 disease will be vanished with this vaccines. But we can take Vaccine as preventive and curative measure.

I am expecting that this Vaccine should be taken. We are doing so much work at ground level in direct contact with public, we don't know when we might get infection from anyone. As not everyone is wearing Mask in Community and we don't know about who have come from

What do you think the government should do to meet your expectations?

For example, All health care workers has been vaccinated and if any of the member develop any serious Side effects, there should be provision of compensation or goverment should also provide compensation for special treatment if required. If any of staff is admitted in hospital, it's expenses should be paid by government and not by staff themselves.

What do you think, when you will be confident/willing to get vaccinated? (What changes can be made in the process?)

Once vaccine arrives and I am sure about its safety, I will take Vaccine immediately.

I have a suggestion for a situation I have witnessed, vaccine is given in front of public. There is a registration desk beside which Vaccine is given keeping a curtain in between. In this situation privacy is not maintained as everything you talk with vaccinator can be heard by person doing registration. For vaccination, there is should be provision of separate room. Registration and vaccination both in same room should not be allowed.

4. What is your opinion on compliance to COVID-appropriate behaviour post-vaccination among us?

There is no guarantee that i will not be infected with COVID-19 disease after taking vaccine. So the ones who has followed Covid appropriate behaviour since date has to follow after Vaccination too. Precautions must be taken like To avoid going to crowdy places, continue wearing Mask outside, special care for children, Drinking hot water and Ukala etc.

5. What is your opinion on the role of COVID-19 vaccine in ending the pandemic?

We are not sure that COVID-19 Vaccination will assure us against COVID-19 disease. No such guarantee is there. So public has to ake care of them themselves in existing situation.

6. Is there anything more you want to add that you were not able to say regarding acceptance and expectations of COVID-19 vaccine in this interview?

There are few over smart people in Community who are not willing to take Vaccine but moreover they are provoking other people not to take Vaccine by spreading rumours about side effects. Such people over smart people who are used to be an obstacle in Vaccination or any other activities should be specially convinced. They must be explained well on pros and cons of vaccination. So they don't provoke other people.

## **Identifier: Healthcare worker 12**

**30 years old, Female, ANM, KG**

1. What is your opinion on getting vaccinated against COVID-19 in the current scenario?

Corona vaccine which has came has already passed 3 trials and has a succes rate of 75-80%. For beneficiary and our family uptill when it is possible anyone does not get corona for that survey conducted for above 50 years and such type of registrations are done to them we will give vaccine. Anyone other than that having Comorbidity to them also we will give vaccine. As they would have low immunity, corona can occur or not occur but by giving vaccine immunity power can increase in them. In short chances of corona infection in them would be less. Any disease would be there, if immunity is good in them than infection will not occur in them.

What is your personal opinion on getting vaccinated against COVID-19?

According to my opinion, previously we would think that common cold is present,I would think that corona like disease is not present, but in reality we came to know that corona is there because common cold which occurs in us and corona is present, there is a vast difference between them like breathlessness, weakness occurs in body that we cannot walk from our own bed.

What is your opinion on the concern surrounding the side effects related to COVID-19 vaccine?

For side effects, yesterday we had given training regarding it as mild, moderate and severe, when patient comes, we have to give common advice and keep them for observation. If any side effects occurs, it does not mean it will occur but if occurs than we have to resolve it.

2. What is your opinion on any misinformation affecting the acceptance of Covid-19 vaccine among the community? What can be done to minimize such rumors?

Such rumours are present like in some small villages vaccine is given which is of no use. I have came to know about rumours like after taking vaccine after 2-3,5 years people will die. This type of rumours are present, so we have to give advice to them and remove rumours. Some are saying that corona vaccine is fake there is no such type of vaccine present. False beliefs are present among them that they do not want to take vaccine than we have to give advice to them. We have to solve their questions by hiving advice to them that if they take vaccine it is for them.

Due to present Covid scenario, with social distancing various drama plays, role play can be done though less number of people is gathered by social distancing. If possible than can be done in aanganwadi, Though children are not coming but 10-10 parents can be called and give advice regarding it to them or by making syringe,video for it can be uploaded on YouTube. All parents who are in our contact or beneficiaries of our area can see video by WhatsApp also.

3. What are your expectations from the vaccine? (take cues from the answers above)

Uptill than it is possible vaccine should get 100% successful for beneficiary and our family, Covid spread gets decreased. We would say that no one gets infection but uptill than it is possible that COVID is decreased so fear can go away from all ,so it would be good for schools and us.

What do you think the government should do to meet your expectations?

Government is giving instructions to what to do according to them we should do and if not possible than we can take health from our staff and we should also give advice to beneficiary. From government they have told that vaccine is to be given but along with that if support is given like medical team along with 108 ambulances.

What do you think, when you will be confident/willing to get vaccinated?

For taking vaccine whenever they will say to us we will take vaccine.

What changes can be made in the process?

At present, we have been told that Covishield vaccine is going to given but when we get it in our hands than we would know which vial is given to us. For that according to me when parents come for vaccination than we will give four instructions which have to be given but also we should give advice that this vaccine is for your health so if any little side effects occur than do not do any type of opposite behaviour against the staff, you would feel problems for 1-2 days but in future it will be good for your health. Otherwise if we tell to public regarding it than other people would run away in fear but we should give advice in advance that side effects if occurred than do not worry and to support health staff so we can give your review to other staff member.

4. What is your opinion on compliance to COVID-appropriate behaviour post-vaccination among us?

For that routine process which are done should be continued like wearing mask, use sanitizer and social distancing.

5. What is your opinion on the role of COVID-19 vaccine in ending the pandemic?

For vaccine, for the first time when vaccine is available and we give it to others after that we can came to know about it. Uptill now vaccine is not in our hands than how can we say about it.

6. Is there anything more you want to add that you were not able to say regarding acceptance and expectations of COVID-19 vaccine in this interview?

For that , uptill it is possible it should be 100% successful, to beneficiary though little side effects are developed we have to give advice to them and support health staff. By giving their review to others of whom survey is done and vaccine is given to each individual so everyone would get healthy rapidly.

Thank you for taking participation in this activity. Thank you sir.

Welcome.

## **Identifier: Healthcare worker 13**

**42 years old, Male, Medical officer, DT**

1. What is your opinion on getting vaccinated against COVID-19 in the current scenario?

According to my opinion, every people should take corona vaccine which is necessary. Though they have suffered from corona or not of vaccine is taken than in future chances to get infection of corona would be decreased. So according to my opinion everyone should get vaccinated. Any Side effects are not present and it is good.

What is your personal opinion on getting vaccinated against COVID-19?

My personal opinion is that vaccine has less side effects and to get vaccinated is imperative.

What are the reasons for your acceptance of Vaccine?

People have thinking that vaccine has side effects so they are not willing to get vaccinated, I have also heard that in vaccine animal fat is present due to which people are facing problem and people have thinking that corona like disease is not present so they do not want to take vaccine and many people are saying that they have developed antibody against it so they do not want it, so people are avoiding it but according to my opinion it is necessary to take it.

What is your opinion on the concern surrounding the side effects related to COVID-19 Vaccine?

Uptill now according to information received there are no side effects of corona vaccine, uptill now no case have came that suggest of side effect of corona vaccine. After Covid vaccine is introduced after that side effects would be known.

2. What is your opinion on any misinformation affecting the acceptance of Covid-19 vaccine among the community? What can be done to minimize such rumors?

Rumours are there like after taking vaccine severe side effects would develop. As I said like animal fat is present and any other serious matter have occur due to which people avoid it.

3. What are your expectations from the vaccine? (take cues from the answers above)

I am only having expectation that all should get vaccine against corona and remain safe from Covid 19. In future this pandemic related to disease would end.

What do you think the government should do to meet your expectations?

In government, those whoever has started working on vaccine is at a great pace and I am requesting to people that they come forward and get vaccinated so that immunity increases and we can fight against Corona.

What do you think, when you will be willing to get vaccinated?

Whenever government will give me first dose I am ready for it so I am going to take the vaccine.

As you stated that people should come forward and accept vaccination for that what should be done? Any suggestions?

For that ISC should be done and those people are not willing to them advice should be given that no side effects are present of vaccine, if you will take it than it is for your benefit. Misunderstandings which are present have to removed for that ISC is needed to be done, marking is done, health worker communication should be present for that.

For ISC any special advice you want to give to us?

For ISC, health worker staff is needed to be trained, Asha worker or aanganwadi worker in there respective areas have to communicate with people, leaders present over there with them sittings have to be done so they can know about it, markings can be done, by organising camps ISC can be done.

At present you would not know regarding vaccination program which is going to be done, so do you want to give any advice so that any improvement can be done in program?

According to my advice, facility is present for it, many facilities are there where 3 rooms are also not available. So it can done that if any facility based private school where vaccination can be done. Over there seperate room can be done where vaccination can be done easily, if it is possible than it is good.

4. What is your opinion on compliance to COVID-appropriate behaviour post-vaccination among us?

After Covid vaccination is finished after than also we have to take corona against precautions like mask, sanitizer, social distancing, we have to give advice to people that corona has not gone away, as much you will follow social distancing it would be good for us. We have to understand that corona is present along with us.

5. What is your opinion on the role of COVID-19 vaccine in ending the pandemic?

There are two doses of Corona vaccine, after taking first dose after 28 days second dose is taken. After taking this dose in you body antibody will develop and your immunity will increase so that you will have less chances to get Corona or Covid-19. Though of Covid-19 infection in your mind would get away.

What is your opinion on acceptance of vaccination against COVID-19 in health care workers?

For that ISC can be done, as I said training can be given to health worker, by taking training information related to Covid-19 can be given.

6. Is there anything more you want to add that you were not able to say regarding acceptance and expectations of COVID-19 vaccine in this interview?

I just want to say that there are no side effects of Covid-19 vaccine, I give advice to every health care worker and people that they should get vaccinated.

Thank you for taking participation in this activity. Thank you sir.

Thank you.

## **Identifier: Healthcare worker 14**

**35 years old, Male, Medical officer, MV**

1. What is your opinion on vaccination against COVID-19 in the current scenario?

As this vaccine of corona is introduced it is a good thing that immunity will increase in people. If all mass or population gets vaccinated than all of them will gain immunity and If any individual gets infected than spread of it would be limited which would be beneficial for current epidemic situation.

What is your opinion on getting vaccinated against COVID-19 in the current scenario?

At present vaccination has started for health worker, as government has stated than slowly vaccine would be available to all individuals so according to me everyone should get it and according to my personal opinion it will be beneficial.

What are the reasons for your acceptance of Vaccine?

As a health worker I am working as a RCHO officer we do immunization in children so I know that we give vaccine to give protection of them. So this is one type of vaccine which is to be given to everybody so in them after having vaccine if they get infected than they would not face any problems or if gets infected than it would not get serious and spread of it would be prohibited so for protection of people this is necessary according to my opinion.

What is your opinion on the concern surrounding the side effects related to COVID-19 Vaccine?

First of all when common people gets vaccinated they have fear this or that type of side effects would develop but in common conditions none of it occurs and normal fever develops or pain at the site of injection might be present. Fever is a good sign of immunity in body, as a health worker we know that immunity is developed which is a good thing, other than that major side effects are not present in our majority of vaccination done. Our vaccination team is sitting with proper preparations so there is no need to worry about any side effects and vacccination should be done.

What's us your opinion on any misinformation affecting the acceptance of COVID-19 Vaccine in health care workers?

Many health care worker are in a state of fear due to rumours present but as a health worker they are already knowing that what is vaccine so they should not believe in such rumours and should take vaccine. They are personally getting children vaccinated so they should not be in fear regarding it.

Have you heard such misinformation/Rumours about COVID-19 Vaccine?

At present in newspaper, there is discussion occurring regarding Pfizer vaccine, around my house there are common people living so they are discussing regarding side effects occurring to kidney, people died due to vaccine such type of many rumours are present but all that things are rumours and it should not be belived by anybody.

As you stated that this types of rumours are present, for awareness regarding it what can be done?

For awareness in routine program we are doing IC which is better, community counselling can be done, mass promotion in media can be done to spread awareness, big doctors give interview, common people from a community or any leader comes forward and takes vaccine and gives interview about it by giving awareness than it is better.

2. What are your expectations from the vaccine?

As early as people get vaccinated and spread of it is prohibited that is the only expectation by me. Vaccine should do its work and good effect shoul be present.

3. What do you think the government should do to meet your expectations?

At the facility where Vaccine is being prepared it should be passed through maximum trials after that it should be launched. During trials if effect of it is less than improvements can be done. For that all the logistics which is necessary should be fulfilled which would be good.

What do you think, when you will be willing to get vaccinated?

I have already taken 1st dose and I will take 2nd dose.

What changes can be made in the vaccination process? Any suggestions?

According to present process vaccination is going on for health care worker and after that for front line worker. After completion of front line worker as early as possible government should make vaccine available to common people which is necessary because it will take a long time to reach to general people.

At present among health worker Covid appropriate behaviour is followed to get protection from corona, masks are wore, hand sanitizer is used, like this safe precautions are taken, after vaccination is done than according to your opinion what can be done to implement these Covid appropriate behaviour ?

According to me it should be followed as this vaccine is new, effect of it would take a long time to come, everyone will get vaccinated will take a lot of time. So uptill that time it becomes sure that effect of vaccine has came, spread of it would not occur uptill than it should be followed.

4. What is your opinion on the role of COVID-19 Vaccine in ending the pandemic?

According to me along with vaccine people should follow Covid appropriate behaviour and vaccine would become 100% or less than that effective. If spread among people would be less due to this vaccine than slowly pandemic will decrease by its own.

According to your opinion this Covid vaccination program is started than which are the challenging factors present in it?

First of all rumours, when vaccine was introduced many rumours have been spreaded amongst health care workers. When this health care program started many health care workers were in a fear of getting vaccinated. At present after program has started they have taken vaccine though many health worker are not still willing to take vaccine. If has also occured amongst front line worker if we become strict than only they are getting vaccinated though many are not ready to take it. So after it starts in general public than many registrations of them will also come. So this is the biggest challenge that how can we get all of them vaccinated.

As you stated about challenges, so to remove such challenges what can be done?

For that first of all we should take care that there should be no spread of rumours and if rumor has been spreaded than that rumour is a rumour which should not be believed by anyone should be spreaded via IC program or mass media or big interviews should be done. As rumour has been spreaded instantly positive news regarding it should be spreaded via media.

In vaccination drive what do you want to say regarding Human Resource availability?

At present along with vaccination routine work is also continued and various health programmes are there where staff is also present so for Vaccination programme HR is less in number though we are trying to keep balance between both vaccination and health programs. In one form of way we can say that HR is less in number.

In vaccination drive what do you want to say regarding logistics?

In logistics there is no such need as syringe and vial is provided in sufficient amount by upper authorities so in that I don’t think so any requirement is needed.

In this vaccination drive what is the role of community perceptions and behaviour according to your opinion?

For that rumours which are spreaded in community is more dangerous than among health care workers because if for once common people would believe about rumours that vaccine is harmful than they would not get vaccinated which is an obstacle for us. To get common peoples behaviour towards vaccine would also be a challenge for us. Uptill today i think so that negative behaviour of people is present towards vaccine and as early as possible improvement is needed to be done.

As you said that negative perception is present among people than to bring it towards positive perception or to develop positive environment in community what special can be done for it?

As we are doing routine program so role play can be done by going into the community, as Covid is present so mass gathering is not preferred so about vaccine it can be spreaded through media , in various community big doctors are present than video of them can be spreaded in their community it would have a more effect on it and we as health worker have seen that wherever big doctors have taken vaccine than staff memebers and other health workers are positive towards vaccine so it is necessary.

In this vaccination program whatever complains are present against vaccine than what is your opinion towards solution of it by system?

For information of it government has made a helpline number of it which is a very good system. Those whoever can call and will get appropriate answer for the question. At local level Aarogya offices are present from there also appropriate answers is been given and after that I don’t think so any complain would remain. Helpline is very useful for it.

Can you give information regarding helpline?

1055 is the helpline number. As for Covid it was made for any troubles faced by people and that surrounding a person’s house Covid positive person is living than they can call on it and gain information regarding it. If anyone has symptoms than where to go for reports can be asked on the same way for vaccination it is same. Rumours related to vaccination. Vaccine should be taken or not all that type of help can be taken from helpline number. So this helpline number is very useful.

According to your opinion on this vaccination program all the enabling factors which are in your favour on that what would you like to say? Which are those factors due to which vaccination program was easy?

From starting phase we had taken private doctors into this so I think so it is a good thing as after that doctors had taken Vaccine than many health workers had developed positive response towards vaccine that vaccine should be taken. In government vaccination many people think that it is conducted by them so we should take vaccine but they have also included private sector so it would develop more positive response according to me.

At present mechanism is present according to universal immunization program than how has it helped/favoured in corona vaccine immunization?

At present universal immunization program is going on in which routine immunization is being conducted than health staff is used to it. Full vaccination session process of how to plan it, to give faulty message, they are trained for it which was beneficial for us as sessions were properly conducted, proper messages were given and safe injection practices is conducted, waste disposal management they were knowing about it due to uit so proper management was done.

At this time of vaccination program, use of IT(information technology) was done. According to your opinion what was the role of it?

Covid vaccination is based on portal as Cowin app and portal is made for it. First of all beneficiary and health care worker had been registered by us on it. Everyone’s mobile number is given on it. SMS are sent on where and when they have to go for vaccination. It is also easy for them that they know where they have to go. When beneficiary go at center and show message of registration than vaccination is done, entry is done and certificate is also generated. So it is a easy and proper process if internet is properly working than no issues are created. After vaccination is done message is sent to beneficiary that they have take vaccine. For second dose also message will be sent where to go and after that certificate would be also generated for the second dose. Due to IT it has become an easy process as message sent also contains their name, number and if any trouble arises than who to contact also is given in message. So it is an easy process due to IT so we do not need to say them orally as by an SMS they would get reminder of the dose.

As we discussed about Cowin portal than any type of issues created while using this portal?

In portal first of all registery is shown. In one form it is difficult for data entry, if a small mistake is present on excel sheet than it does not accept it. Many times it has occured that while uploading staff data list due to an error it is not been uploaded than on field it comes to know that any beneficiary has given detail but when they reach at vaccine center registration is not been done than it is bit of challenge for us. As counselling of them is needed to be done that registration of them is pending. Other is internet is not good, in all parts of india sessions are going on so portal is working for it and servers become slower in speed so time of session increases to 15-20 mins instead of 5 mins.

5. Is there anything more you want to add that you were not able to say regarding acceptance and expectations of COVID-19 vaccine in this interview?

No. I have said everything.

Thank you for taking participation in this activity. Thank you sir.

Thank you.

## **Identifier: Healthcare worker 15**

**51 years old, Male, Medical officer, RKS**

1. What is your opinion on vaccination against COVID-19 in the current scenario?

Covid vaccine is very safe and effective. It’s efficacy is around 70-90% as per data given. There was a fear among people regarding side effects of vaccine than we have given vaccine to 2600 healthcare workers in bhavnagar, not a single AEFI case has been detected by me and not a single complain has been given to us in the system. Common cold and pain is present but that is also side effect of all the other vaccines so there is no such type of major side effect present.

What is your opinion on getting vaccinated against COVID-19 in the current scenario?

Vaccine is safe, no side effect is present as per data and health care worker related to public health are supportive and acceptance of them is approximately 90%. Other than that aanganwadi worker or others who are not directly related to it but are along with us in work in them not enough response is obtained.

What is your opinion on the concern surrounding the side effects related to COVID-19 Vaccine?

No, there is no such type of thing. Uptill tomorrow we have completed vaccination of almost 2600 and no major Aefi or side effect is been detected. So now there is an evidence that this talk is not that much important about false rumours and if an individual according to their body 1 in a million can develop that I am not denying but in routine we are not observing it.

2. Overall when vaccine was introduced at that time What are your expectations from the vaccine about its efficacy so that it’s acceptance would have been increased?

In that from starting unnecessary rumours were created which were spreaded by media and social media due to which apprehension was more in beginning. But as we continued doing vaccination at present acceptance of vaccine is more than in past and people are taking vaccine.

3. What do you think the government should do to increase acceptance and expectations of vaccine?

Government has done enough efforts behind it even politically they have given good assessment. From our department query have been solved by giving proper guidelines for it. Whenever new things are introduced in every thing hestitation is always present like when we had conducted vaccination campaign many challenges had been faced by us but after overcoming them we had successfully completed 100% vaccination in that also.

4. What is your opinion in compliance to COVID appropriate behaviour post vaccination in Health care workers?

No that should be followed because after getting first dose of vaccine there is a gap of 42 days for second dose and after 12 days of first dose antibodies are developed inside the body to develop immunity so this behaviour should be maintained.

At present in common people to get protection from corona, masks are wore, frequent hand washing and social distancing is present like this safe precautions are taken, after vaccination is done than according to your opinion what can be done to implement these safe precautions ?

5. What is your opinion on the role of COVID-19 Vaccine in ending the pandemic?

Now we can see that in our city and state it has already came under control and now what will happen depends on the future. According to me now whatever little bit of pandemic is left will get over in short period of time.

Covid 19 Vaccination program is already rolled out. Any problems you as a MOH have faced uptill now?

Yes there was a challenge regarding vaccination center having 3 rooms is not been available and wherever necessary center required according to gov guidelines had been created and in the beginning those who are not related to our health department they were resistance for us and a little bit is still present. More challenge have not still come and as per the target we are ahead of it. In reality there is little bit of problem in Cowin software and those duplicate entries done for them there should be an option for removal of it which are done by mistake.

In Covid vaccination program problems related to any Human Resources?

No there is no such issue present. We have enough and sufficient number of staff members present.

In this vaccination drive any role of community perceptions and behaviour according to your opinion?

No it is good and in this aspect private doctors are demanding to get vaccinated along with their family members. Even many association are demanding to me like yesterday MR association had came and requested that sir you should provide us vaccine as we are health related. So demand of it is increasing compared to past which is good.

In this vaccination program whatever complains are present against vaccine than what is your opinion towards solution of it by system?

Whatever complains are presented like there name was left out or any type of trouble is faced by them than by taking our own interest we instantly solve their problem.

According to your opinion on this vaccination program all the enabling factors which are in your favour on that what would you like to say? Which are those factors due to which vaccination program was easy?

Pandemic was present, many people were in trouble. Enabling factors like ISC was such a good thing. Information related to safety was given. All this type of communication developed between people which can be stated as enabling factors. And even our health staff and vaccination center gives advice that vaccine is safe and tells people by giving their contact number that whatever side effects develops you can freely call to me. That builds the confidence in the community and due to this slowly it became our helping factor.

At present routine immunization program is going on than how has it helped/favoured in corona vaccine immunization?

Yes that was already present as our staff is giving routine vaccine so they are aware about vaccination due to which more training was not needed by staff as they were already trained so they learned it in a fast manner and completed it successfully. And for it routine immmunization is not hampered for that we conduct 3 days a week routine immunization (Mon,wed and Fri) and tue, thrus and sat we conduct Covid vaccination. So without any hurdles both are working simultaneously.

At this time of vaccination program, use of IT(information technology) was done. According to your opinion what was the role of it?

At present it is totally IT based otherwise we cannot give vaccine to an individual. We have to first register name in Cowin portal after that SMS is sent after that we verify. After verifying with Aadhar card or any document we give vaccine to that individual. In the software chances of doing anything wrong is negligible.

Sir as you said hesitancy and resistance is still present in community or health care worker so what can be done for it?

Purs healthcare worker of our department in Phc, chc or urban health center are not creating any resistance but aanganwadi workers and some other workers are creating a little bit of resistance. For that their authority and commissioner has given instructions to

them and given advice to them and we have personally taken vaccine, our doctors have taken it, all health staff which are working with us has also developed confidence in them and I think so we would also be successful in that aspect also.

Sir you stated about IC, can you please explain more about it?

IC is present at our vaccination site, by state government in newspaper, media, electronic media IC is given. In a big amount by mass media IC has been sent and in our staff by IPC(interpersonal communication) to beneficiary it has been given as an advice. So by These way it is benefitted.

As you stated that issues are there with Cowin app than what type of corrective steps can be taken for it? In future if any new program is made than how can we improve IT sector?

In the beginning we had faced problem in registration and uploading of information and session creation problem. But now that problems are not there it has been solved. In beginning whatever work we are doing it was a unforeseen problem.For the First time this type of app was made to work at whole India level so a little bit of trouble was there but now they have made improvement. Now there are no problems in Cowin app and portal. So now when we would include mass number of health worker and front line worker along with common people than past problem will not arise again.

Sir as you said support of private practioner was good , do you want to add anything?

Yes it was very good. Presidents of IMA and all of them had taken vaccine, they had done IC in a productive way, all of them have taken and nothing has occured to them. They have given good support.

6. Is there anything more you want to add that you were not able to say regarding acceptance and expectations of COVID-19 vaccine in this interview?

No I have done enough of the talk and hope so we can get all of the individuals vaccinated. Little bit of resistance is present which is seen by others as an apprenhension. By seening others that nothing happens than they will come and get vaccinated.

Thank you for taking participation in this activity. Thank you sir.

Sir do you want to add something else?

Problem over here is that those who have come from lower study background committee like aanganwadi worker collect newspaper from various places and they print this type of negativity in newspaper. Negativity is rapidly spreaded by social media like WhatsApp. Some of these people are getting ready for spreading misinformation regarding health worker. Thea type of things are occurring in government sectors due to aanganwadi workers otherwise there is a big line in private sector for getting vaccinated and their registrations. There is no problems among people over here, I do not take attention towards all this type type of rumours as all over here willing to take. After taking vaccine it is common that fever and body pain would occur, after having vaccine one does not get paralysed. Those individuals who have taken vaccine are working in Opds and over here but a little of such problems will always remain over here.

## **Identifier: Healthcare worker 16**

**33 years old, Male, Medical officer, VK**

Q: Were you or any of your family members Infected with corona?

No.

Q: What us your opinion about vaccination against corona?

We have seen all the time that vaccine can be a thread to hold on in these difficult times. Whole country is fighting it rather whole world is fighting corona, to get rid of it we have got the vaccine.

Q:Opinion on taking the vaccine?

Everyone should take the vaccine.

Q:What are the reasons for your opinion?

From first case in Bhavnagar to vaccinating policemen yesterday I have seen it all, people getting infected and even people dying and other people burying those people. When you have got a change to protect yourself and your family members everyone should definitely take the vaccine. There is no reason to take this vaccine.

Q: Opinion about side-effects of vaccine?

I have heard, as a health professional, we read news papers and people also come to us about it there might be some side effects but serious side effects are very uncommon. And if we see four people out of 5 eat pan mava smoke cigarettes etc they don’t think about that and vaccine is a good thing so people should not think more about side effects of it.

Q: Have you heard any discussion about side-effects effects from vaccine?

Many people tell that people get sterile after taking vaccine and even people die after taking the vaccine, heart, kidney gets damaged. The vaccine is not effective or people get corona after taking the vaccine. These are all misinformation I have heard.

Q: Mis information in health workers?

This occurs rare that there are misinformation is there in health workers. But after all health workers are humans and there are many female who fear injections and anxiety about it. If any health worker refuses to take vaccine this might be the reason i.e. fear of needle or anxiety. Refusal is not due to mis information, I believe so.

Q:Have you heard about any other mis information?

No nothing new I already mentioned above.

Q:What are your expectations from vaccine?

I know vaccine is effective and I have no complaints about it.

Q:When will you be ready to take this vaccine?

I am ready to take this vaccine when my turn comes.

Q: Are there any changes that can be made in the vaccination process?

People who need it most should get it first.Vaccination is going on in large scale and people in high risk, health workers , police, teachers should get it first as soon as possible.

Q: Role of CAB after vaccination?

Should follow covid appropriate behaviour as there are proof that only after second dose person develops immunity against it but it is not so in 100% of cases, depends on efficacy of vaccine. It is not possible to vaccinate all people in short time so if we follow all things people will see us and they too will continue following covid appropriate behaviour.

Q: Role of vaacine in ending covid pandemic?

As the gong is striken at Victory in kurukshetra, similarly bells of victory over covid have started ringing. Very soon there will be end to covid.

Q: Challenging factors in vaccination?

Main challenges were misinformation. Such misinformation are there in all the new programs that this not be taken or this should not be done. When we go to vaccinate people they ask us have to taken the vaccine first. Why are you giving vaccine to us, they don’t know whether we have taken the vaccine or not.

People lack awareness about the truth, that is the biggest challenge.

Q: What can be done to overcome misinformation?

As much as possible IEC should be done. Doctor, leaders, politicians,or people doing social work, IEC should be done through all these people then it would me more effective.

Q:Opinion about Availability of Human resources ?

We have vaccinated more than 3000 people in Bhavnagar city but we haven’t face such issues. We are running multiple programs such as polio then IMI will come soon and all routine health programs have started and mostly we haven’t faced any problems in manpower.

Q:Logistics?

The biggest plus point about logistics is all the things we need for other vaccines can be used in these vaccine too. We don’t need anything new such as special type of refrigerator, or special Needle etc. For these vaccine all needed logistics are same as others which we use routinely. We have got everything adequately.

Q:Community behaviour in covid vaccination program?

There are two points. Relatives of health care workers are aware as health care workers have already taken the vaccine. And there are other people who don’t know anything and hence they do not oppose. But some groups oppose and it feels like they are born to oppose all things.

There are not much challenges. People are getting willing more and more, we do IEC in social media and all. As first health workers are vaccinated it has created trust in people that those who worked in corona have received vaccine to it is safe.

Q: Grievance redressal ?

I don’t think people knew that there are 13 urban health centres in Bhavnagar. There are UHCs in posh area and slum area too.

We have shared phone number of UHCs and even mobile numbers of medical officers in newspapers and people know where to go for corona related or health related problems. There are helpline numbers of state govt and Central govt too, 104 and 1075 and so people can call those numbers and solve their queries.

Q:Enabling factors ?

We have started vaccination with health care workers and front line workers, these are those people who have worked amongst corona positive people, so they understand the importance of vaccine. Vaccinating these people went easily and so side effects are seen and even if there is spread of some misinformation then we have addressed it on emergency basis, such as doing IEC, forwarding true information on whatsapp and facebook. In bhavangar city we are doing vaccination since two months and I believe that there is less spread of misinformation.

Q:Role of UIP in corona vaccination?

System used in UIP are to be used in this vaccine too. Same technique is used for IM injections, same temperature used for storage. There is no new site of vaccination, it is to be given at arm and we have vaccinated numerous children in other vaccines. Storage of vaccine in vaccine carrier and transportation to session site is similar to what we do in other vaccines, nothing new, our system is well rehearsed about all this, so there are no problems.

Q:Phone number of UHC and staff were given to public, are there any Convenience/difficulties faced in that?

Our centres are divided area wise and so it is easy for people to know where to go and whom to contact so they can contact in case of emergency. Our helpline of 104 is best, suppose you call it now and say that you have fever cold cough, I will get that information in 15 minutes, I will get a mail and in the next minute I will get a call , I will inform the concerned center and with in two hours your follow up will be taken. That is the strongest point.

Q: What ere the difficulties?

Health care worker is a human too, we don’t have so many people that we can change the staff round the clock at every 2 hours, so many problems occurs, in the beginning people had to work for 18-18 hours in a day, patient are to be shifted at night 2 AM 4 AM and next day you have to be present at 9 am in the morning, many a times while eating we get call to take follow up or do rapid test of this patient. There are people who do not understand but as a health care worker we counsel them and then they understand so problem is solved.

Q: Role of IT in coid vaccination program?

Vaccination is going on based on online system based, we have entered data in the portal already, we plan session according to that data suppose we are given target of 100 people in a session then we get list of names of 100 people from the online portal. Only people in that list can get vaccine. If today is your turn to get vaccine then you will get a message a day before that you have to go at this place and at this time to take the vaccine. Suppose you got vaccinated now, within 10 minutes you will get message that you have been vaccinated and who administered the vaccine to you, such is the system and it is good.

Q: Others benefits of use of IT in vaccination?

I my experience people with this it is easy for people to believe in us, we vaccinated senior doctors and they were happy that govt is doing so much for them and their perspective changed. Many of my friends and senior doctors said that they never thought that govt will do this much. People come to us in Lamborghini for getting the vaccine. They can get vaccinated anywhere they have got high contacts but they come to us and they are happy when they go back.

Q: Please tell us more about 100 people per session site and number of people coming to get vaccine?

When we plan the session, one vaccinator can vaccinate 100 people and message is sent to all those 100 people, they do come but names of many people are not registered, suppose I get the message but my colleague does not get message, they also come to session site and inquiry about it, we provide solutions to their queries and plan for vaccinating them too in future.

Q: lastly would you like to say anything more about Acceptance and expectations from vaccine?

For acceptance I will say this, people opposing vaccine should come out of it and get vaccinated if you are eligible. I will say only one thing, thank god that you are alive to take this vaccine, am not saying this for the interview but I really feel this, that people should be thankful to god that they are alive to get this vaccine and should take it.

Q: Any suggestions/changes that can be made to cowin online portal?

There are not issues of network but there are sometimes problems in cowin portal, cant log in on time, or not able to upload data on time, but it is going to be there everwhere, no matter how good you do there are always going to be some problems, nothing new in that.

# Community Participants

## **Identifier: Community participant 01**

**58 years old, Male, Tailor, NM**

1. What is your opinion on getting vaccinated against COVID-19 in the current scenario?

A. people have perceptions, what if side effects or any problems occurs by taking vaccine. What if something new happens to us. people have fear regarding this. According to my estimate there is nothing to fear. People have never taken such vaccine so there is fear in minds of people. So, people won’t be ready instantly. Those people infected with corona are going to take the vaccine they feel that they need the vaccine. Government has to come up with some programme that what are the benefits of the vaccine, how it will affect our future such information should be spread by making banners and other things. Then only this society will know. If they only give ads in newspaper that vaccine should be given then how will people know. In our country there are so many illiterate people, needed level to literacy is not there. For a time being people did not know about corona still some common people tell “what is this corona? it is just hoaxes aired by people.

What are the fears in the people?

If vaccine is taken, if it benefits people then good but if some reaction occurs or some other problem occurs such fears are there in the minds of people. People don’t know whether vaccine is effective or not. First politicians should take it. they are giving it to common man but why aren’t they starting with the authorities.

As you said information is not reaching people, what can be done for it?

In Bharat people don’t have the habit of reading newspapers, so by using whatsapp, banners at public places such as post office, railway station, on roads or on big temples then awareness will spread in society about the vaccine and what are its advantages.

What is your opinion about taking corona vaccine?

Two people form my family have been infected with corona, so I believe I should take the vaccine

(a) What are the reasons for your answer (Willingness)?

Its for our safety, I understand that much. If we go out then at least for some time we will be safe from corona and corona will not easily affect our body.

(b) What is your opinion on the concern surrounding the side effects related to COVID-19 vaccine?

There are opinions of different people. If some side effects occur in some one e is going to say side effects occurs. I have not taken the vaccine so how can I know. So advertising about this is not favourable for society and country.

(c) What is your opinion on any misinformation affecting the acceptance of Covid-19 vaccine among the community?

if awareness is spread only then people will be aware about this or else, they won’t be willing. They will run away from vaccine; common people too will stay away. Those who need the vaccine i.e., the people gathering in the markets. Right now, there is festival of shankrant, see how many people are there, no one fears corona.

It is in todays newspaper that vibhavriben has done opening and no social distancing was followed even by the government and government says keep social distancing.

You talked about hoaxes what are some hoaxes that you herd about corona vaccine?

There might be fear in people and if some side effects occur after taking the vaccine then where to go and whom to contact there is no awareness about that. I have also herd that somebody had taken the vaccine and side effect occurred and the person has registered a claim against the company, I have heard some one has demanded 3 crores in Pune. This is not appropriate. This is not favourable to do this against the company that developed vaccine according to me.

2. What are your expectations from the vaccine?

Vaccine is good. Almost 10 months has passed since corona has come to India and after this much time if vaccine is developed and it reaches common people then it is benefit of people and fear of getting infected with corona will not be there. My expectations are that, vaccine reaches to all people.

3. What do you think the government should do to meet your expectations?

I already said before that government should put banners at post office, railway stations, temples and other public places. Also, to reach common people they should print pamphlets. It is already coming in news. There are so many other channels, advertisements should be spread through them free of cost for government. Things should be done so that this information reaches common man.

(a) What do you think, when you will be confident/willing to get vaccinated?

A. I need to take vaccine because of my age. Whenever I am told I will take the vaccine.

(What changes can be made in the process?)

Already ad good plan has been prepared by the government for billions of people. For all villages and cities good plan has been prepared and government should be congratulated as this much things are being done by them.

4. What is your opinion on compliance to COVID-appropriate behaviour post-vaccination among us?

I have read in newspaper that even after vaccination we have to follow all this, wearing masks, handwashing etc. It is necessary to be so.

5. What is your opinion on the role of COVID-19 vaccine in ending the pandemic?

A. Government has sent so much; all this companies has worked hard and developed vaccine and if it reaches all then it is good.

6. Is there anything more you want to add that you were not able to say regarding acceptance and expectations of COVID-19 vaccine in this interview?

No nothing else, I have already said above. Awareness should be spread by using banners and other things so that people can understand about this and then they will be ready to take vaccine.

## **Identifier: Community participant 02**

**54 years old, Male, Employee, MB**

1. What is your opinion on getting vaccinated against COVID-19 in the current scenario?

A. Vaccination should happen and all should take the vaccine.

What is your personal opinion about taking the vaccine?

A. Should take it.

(d) What are the reasons for your answer (Willingness)?

Because it offers protection for the future.

(e) What is your opinion on the concern surrounding the side effects related to COVID-19 vaccine?

A. Side effects occurs rarely to someone and if all the doctors of India are taking the vaccine then there cannot be any problem with the vaccine.

(f) What is your opinion on any misinformation affecting the acceptance of Covid-19 vaccine among community?

A. All the opposing companies can be spreading misinformation so that the vaccine making company is not benefitted but we should not believe any hoaxes and we should take the vaccine.

Have you heard any such misinformation?

Many a times it has come in newspaper that at a place someone has died after receiving vaccine. There might be some other reason for that, I don’t believe that it has occurred due to vaccine as scientists have worked very hard and also PM has taken personal interest so there cannot be such problem in the vaccine.

2. What are your expectations from the vaccine?

A. We should not get infected with corona after taking the vaccine, that is my only expectation.

3. What do you think the government should do to meet your expectations?

All the things being done right now by the government are alright.

(b) What do you think, when you will be confident/willing to get vaccinated?

A. I am ready when ever the vaccine is to be given.

4. What is your opinion on compliance to COVID-appropriate behaviour post-vaccination among us?

A. If it is not necessary then at least wearing masks should not be continued. Washing hands is beneficial everyday and there are other benefits too from washing hands but there are difficulties with wearing masks.

5. What is your opinion on the role of COVID-19 vaccine in ending the pandemic?

It seems it might come under control because of vaccination. It has already decreased much but will be further controlled by this.

6. Is there anything more you want to add that you were not able to say regarding acceptance and expectations of COVID-19 vaccine in this interview?

A. No, it just that all should take the vaccine, I believe that personally. Vaccine is good and all should take it.

## **Identifier: Community participant 03**

**56 years old, Male, Govt. employee, SS**

1. What is your opinion on vaccination against COVID-19 in the current scenario?

At present according to my knowledge 2-3 vaccines of corona have been introduced in India like of Serum institute of Pune at present most of it is given and another from south India of Bharat biotech which is been used in various states of South. Vaccines given in Europe I think so has more fatality rate and side effects are more. Vaccine given in India is not in that much severity according to me. Uptill now vaccine is given to medical staff and frontline workers so very less % number of people have been given vaccine. On this basis we can say that adverse reactions in India is less in number but after total completion of 2 doses any reaction would occur or not which is not yet final. At present to get vaccinated or not is also double minded thing for me because my health is a little bit substandard as pacemaker is kept in mine body, am suffering from asthma and pleurisy. So according to me after a proper study and research along with proper precaution I will decide to get vaccinated or not.

What is your opinion on the concern surrounding the side effects related to COVID-19 Vaccine?

In a very short time vaccine was introduced so a long study would have been conducted regarding side effects of vaccine in our and other countries also. But other vaccines were present in past other than corona such vaccines might had side effects which might not had been in notice to everyone but vaccine having lesser side effects would be beneficial to us.

Which type of side effects have you heard of? Can you state it?

In very less percentages many cases have become fatal, other side effects like facial paralysis

occurs, other common side effects of vaccine like common cold, fever, body pain this type of side effects have been heard by me.

As you stated about side effects, from where have you gained this information?

In most cases via the internet like from google.

What's us your opinion on any misinformation affecting the acceptance of COVID-19 Vaccine in common people?

The information given in newsapaper or WhatsApp may be wrong but from the information on the internet have been given by authentic institutions so it can be considered reliable.

Have you heard such misinformation/Rumours about COVID-19 Vaccine?

Yes I have heard about one thing that big medicine manufacturing companies and international companies have created this big fear about corona, in reality there is no such type of corona present so no need to worry about it. This might be a rumour.

2. What are your expectations from the vaccine?

Masks should be eliminated. Vaccine should be having more efficacy than it would be good. Side effects shoul be maximally minimum or should not at all present than it is good.

3. What do you think the government should do to meet your expectations?

For our organisation which is of service class for us, self paid in private hospitals to take vaccine option should be given. We can get early vaccinated as government is giving to government servant or to them who is necessary to have vaccine. In parallel to us who are capable to take vaccine by our own expense in private hospitals if government gives option than it would be good so that much mass production should be present.

What do you think, when you will be willing to get vaccinated?

In feb ending or upto March 15 because uptill than we would come to know about average

number because some people would have completed 2nd dose and nothing would happen to them.

At present in common people to get protection from corona, masks are wore, hand sanitizer is used, like this safe precautions are taken, after vaccination is done than according to your opinion what can be done to implement these safe precautions?

According to me when in a big amount of population gets vaccinated, herd immunity vaccinated individuals of Indian population would have become approximately 50-60% after that vaccine should be taken according to me.

4. What is your opinion on the role of COVID-19 Vaccine in ending the pandemic?

Anything other than vaccine cannot end it. Vaccine is necessary.

5. Is there anything more you want to add that you were not able to say regarding acceptance and expectations of COVID-19 vaccine in this interview?

At present in Indian states, there is no option for vaccines in all states , suppose I want to take vaccine over here than there is no option available plus according to me vaccine which is made in united stated by Morgan which is taken by nasal route which is Moderna. Efficacy of Moderna vaccine is more and is easy to take and I think so only one dose is only there. So if that option is available than it is more good. So we can personally decide that this vaccine should be taken by me.

Thank you for taking participation in this activity. Thank you sir.

## **Identifier: Community participant 04**

**52 years old, Male, businessman, AM**

1. What is your opinion on vaccination against COVID-19 in the current scenario?

A. Vaccination is a type of ladoo made of wood if we eat or not we would have regret of it because many people after having vaccine have died and some people have not died after having vaccine so according to me vaccine should not be taken. I am personally as a social worker have been in contact with one patient everyday and I have given service to many patients and also carried many dead bodies of patient. I, Haribhai kalva, hirabhai have done this services since whole lockdown and at present if any people is in trouble than we stand besides them. I am not having any type of fear against corona but I am having fear of taking vaccine because I have read in WhatsApp and newspaper that many people have died and have developed side effects. Those who have not taken they do not develop any illness so according to me by taking this vaccine is not beneficient.

What are the reasons for your not acceptance of Vaccine?

Everyday in WhatsApp there are news that a common man died after having vaccine. Though I don’t know that antibodies are formed or not, like as a patient by taking treatment recovery can be made which is good although by taking vaccine due to side effects people die due to it. So any person would not die intentionally by self.

What is your opinion on the concern surrounding the side effects related to COVID-19 Vaccine?

Yes that is really true. There was a person from baroda who was in medical profession working in hospital, took vaccine in same hospital and died over there, it was a multispeciality hospital though doctors couldn’t treat him and like that many cases seen in ahmedabad, baroda and surat which I have read in WhatsApp that this case is presented and not cured otherwise corona patient in lakhs of people have been treated from corona. Death rate from corona is only 2% at present and death rate of people taking vaccine is 4%.

The information you gave currently, from where have you heard all of this from?

At present media has came forward a lot like of WhatsApp and news channel that any news comes first before it has occurs. Everyday news come that these number of people lost eyes, these people died. Recently my friend relative from Ahmedabad took vaccine and lower part of his body got paralysed, he was a good businessman. So these type of side effects are present by taking vaccine which is correct.

What's us your opinion on any misinformation affecting the acceptance of COVID-19 Vaccine in common people?

Government is not having complete information regarding it uptill than how can people will come to know like vaccine is introduced but government is unable to explain people that by taking vaccine people will not die, many people have died Government should place a panel of doctors while giving vaccine so if any side effects occurs than they should treat over there but no such type of thing present. We would be given vaccine on the road than how could that be done.

2. What are your expectations from the vaccine?

Our expectations are good that after having vaccine people should not suffer anything. Various famous ministers have developed corona after taking vaccine so I do not feel like that vaccine has came. At present people are misguided by telling them about vaccine. If people who have taken vaccine is told to take corona positive patients to take them to hospital than no one would come and if like me those who have not taken vaccine is told to take corona patient to hospital or burial than hundred people would be ready for it because they have faith in god. After taking vaccine people feel fearless that nothing will happen to them but in reality side effects have developed in them like fever, cold, headache , body pain. After 1 whole month of taken vaccine they still suffer from leg pain and does not get relief from pain.

3. What do you think the government should do to meet your expectations?

For giving vaccines, government should construct multispeciality hospital for it, over there proper staff should be there, those who take vaccine 3 days of complete bed rest should be provided and after that person goes home than it would good. Now if you give vaccine to person on road and if he dies than people will think that he died due to vaccine. In reality people are not dying due to vaccine according to government but they are dying which is observed than how can we trust it. As I said 3 days of treatment should be given in multispeciality hospital and after that vaccine is given than real result would be obtained.

What do you think, when you will be willing to get vaccinated?

As I said if we are good in health and no one is ready to die intentionally as after taking vaccine many people are dying and if we develop corona than treatment is already given and we would be cured. For once I have suffered from corona and I have been treated from it. So I do not feel to take vaccine and by staying at home die and people to talk about that he was good in health and took vaccine and died tomorrow. If corona develops than we have to go to hospital but by taking vaccine and to go to hospital is not good according to me.

4. At present in common people to get protection from corona, masks are wore, frequent hand washing and social distancing is present like this safe precautions are taken, after vaccination is done than according to your opinion what can be done to implement these safe precautions?

Precautions are good and it should be implemented. It is not for good people as to wear masks is a money making business for government. Mask should be weared by positive people so it would not spread from them. This Rs2 mask is weared by people on the roads to prevent spend of Rs200-500. Mask is not weared by them to think that it would be good for them to wear masks.

5. What is your opinion on the role of COVID-19 Vaccine in ending the pandemic?

At present there is wastage of money by government and people are being misguided other than that nothing is there. Corona vaccine is there which is taken by many ministers who also has developed corona after taking it. So I do not feel that any vaccine has came of it. This is just for wasting of peoples money and to increase of intake by government and to misguide people. So no people have trust in it. No country is there which has become cured due to corona vaccine so in our country I do not feel like it can become corona free due to vaccine. By freedomly people should understand and can give fight to corona otherwise I do not face trust on vaccine.

6. Is there anything more you want to add that you were not able to say regarding COVID-19 vaccine in this interview?

About this vaccine doctors have developed corona after taking vaccine and many ministers have developed it. Without taking vaccine I, haribhai kalva, and our team was there have taken people since the starting of lockdown to hospitals, those people who died we have taken them to burial we have not developed anything. Without taking vaccine we have trust on us and our god. We do not have trust on government and we do not want to take vaccine for it.

Thank you for taking participation in this activity. Thank you.

## **Identifier:** **Community participant 05**

**52 years old, Male, Govt. employee, MK**

1. What is your opinion on vaccination against COVID-19 in the current scenario?

For this pandemic, at international level for the control of it, this vaccine availability is awaited but after more and more trials and it should be made user friendly and fear in people also gets away, publicity should be done than it is good.

What is your opinion on getting vaccinated against COVID-19 in the current scenario?

According to me we should wait and watch, after couple of tests and trials on people after observing side effects and such things after that I would see.

As you stated that fear should get away in people, so what can we do to remove fear from people?

For removal of fear means in maximum number of people gets vaccinated of common community, awareness is spreaded among common people like no side effects are developed after getting vaccinated and by after observing it people will come for vaccination by themselves that vaccination is necessary.

What is your opinion on the concern surrounding the side effects related to COVID-19 Vaccine?

From media I have came to know about various side effects regarding it but physically I have not observed or listened about any side effects in people.

We discussed regarding side effects, which types of side effects have you came to know or have listened about it?

No, I have not listened much about it but common things like fever, pain in body, etc which is in common.

2. What's us your opinion on any misinformation affecting the acceptance of COVID-19 Vaccine in common people?

As this pandemic is huge, people are going to discuss regarding rumours and rumours are going to be present among people but slowly people will understand regarding true things and people will observe effectiveness of this vaccine and automatic it will be successful.

Have you heard such misinformation/Rumours about COVID-19 Vaccine?

No, I have not heard any type of rumour regarding it.

3. What are your expectations from the vaccine?

By vaccination, those people who are trapped in their house since last 1 year in big countries and in our country corona is much under control but people are having mental fear like if disease might appear again. My neighbour who is living in Kerala, over their at present condition disease has appeared again, in our Gujarat condition is under control but over there movement of people is more. Yesterday,I came to know about it condition over there is very bad as disease has appeared again. So if effective vaccine is introduced than it would get minimised. Like in past when measles was eliminated in the same way this disease should get eliminated and people will get rid of wearing masks which is an expectation that I am having.

What do you think the government should do to meet your expectations?

Government is already taking steps but more steps like a celebrity comes forward and their vaccination programme is organised than people will get more aware regarding it and fear will go away from people.

4. At present in common people to get protection from corona, masks are wore, frequent hand washing and social distancing is present like this safe precautions are taken, after vaccination is done than according to your opinion what can be done to implement these safe precautions ?

According to me, we get rid of mask as it is an obstacle for people, frequent application of hand sanitizer like that precautions should go away slowly which is an expectation by common people. All people have expectations that this should go away.

5. What is your opinion on the role of COVID-19 Vaccine in ending the pandemic?

Main role in ending pandemic would be done by vaccine. Though any thing would have successful and unsuccessful part of it. But after long time like vaccines of measles and polio is present, in the same way vaccine is the best way for it according to me.

6. Is there anything more you want to add that you were not able to say regarding acceptance and expectations of COVID-19 vaccine in this interview?

Among people for awareness, various programs should be conducted and selfless doctors like you give more and more information to people by various mediums, than more and more people will come forward and fearlessly they would come forward according to me.

As we discussed regarding awareness, any special advice you want to give which would be more useful for this program?

Mediums like WhatsApp are present but more rumours are spreaded through it but information can be spreaded fastly to people according to me and by various media’s awareness can be spreaded.

Thank you for participating in interview. Thank you sir.

## **Identifier:** **Community participant 06**

**56 years old, Female, Employee, GS**

1. What is your opinion on vaccination against COVID-19 in the current senario?

I am having too many hopes, as soon as vaccine is available we can live without any fear and if I would get an early chance than I would get vaccinated so that my family members would not get infection from me for that I will take precautions.

What are the reasons for your acceptance of Vaccine?

Everyday I am sitting on a cash counter in my job, everyday I am in contact with around 125-150 people and currency notes are received from which location that I don’t know, so according to me maximum contamination is spreaded through notes, so everytime I am in fear that due to me my family would not get infected due to it. So I wish that as vaccine is introduced after that I will get vaccinated.

What is your opinion on the concern surrounding the side effects related to COVID-19 Vaccine?

Any vaccine would have its side effects. When we take child for vaccination than doctor states that child will have fever than that is possible. So I do not feel it terrifying.

2. What's us your opinion on any misinformation affecting the acceptance of COVID-19 Vaccine in common people?

According to me instead of accepting news on tv or newspaper, to actual ask doctor in hospital will clear all our doubts regarding it. So instead of accepting rumours, we should ask to our known doctor who is meeting patients every day by asking them our doubts will go away or get cleared.

Have you heard such misinformation/Rumours about COVID-19 Vaccine?

No, I have not heard any rumours or if such rumours are there than I am not taking interest in it.

3. What are your expectations from the vaccine?

According to me whichever vaccine we take we should get assured that for 6 months or 2 years we would not get infected. It could be possible that after 2 years booster dose is needed to be taken that I agree but there should be an assurance from vaccine that for a particular period of time we would not get infected.

What do you think the government should do to meet your expectations?

Whenever vaccination is conducted by government in government hospitals there should a assurance certificate with vaccine that for a period of time you will get protection from infection which should be written on certificate like 6 months or 2 years for a stipulated period of time if such certificate is given by government that that’s well and good.

Other than this do you have any expectations from vaccine?

No, it is a human body and infection can be received anytime or anywhere but if a little assurance is there than it is good for vaccination.

What do you think, when you will be willing to get vaccinated?

If I would be told to get vaccine tomorrow than I am ready for it because I am not having any doubts and I also think that those persons who are in medical field like I know many people who are doctors who are doing work everyday in hospitals they tell us that they have got vaccinated than it means that we are much safe. As they have got vaccinated, I am ready for it.

4. At present in common people to get protection from corona, masks are wore, frequent hand washing and social distancing is present like this safe precautions are taken, after vaccination is done than according to your opinion what can be done to implement these safe precautions ?

Uptill when all people around us would not get vaccinated such precautions should be taken care of because it is necessary for safety of all of us.

5. What is your opinion on the role of COVID-19 Vaccine in ending the pandemic?

I am having many expectations that after vaccine pandemic will get in control and it will progress towards ending of it for that it is compulsory that a very big mass of population should get

vaccinated,2-5% or 15% people vaccinated would not get preferred, atleast half or more than that population would get vaccine than this disease will get ended.

6. Is there anything more you want to add that you were not able to say regarding acceptance and expectations of COVID-19 vaccine in this interview?

According to me if doctor is saying that they have been vaccinated than who could be more faithfull person than them. When hospital doctors are saying publically or by WhatsApp or Facebook that they have taken vaccine that is most faithfull thing and people will get ready fast for vaccination.

Thank you for taking participation in this activity. Thank you. Thank you and have a nice day.

## **Identifier:** **Community participant 07**

**57 years old, Male, Employee, JM**

1. What is your opinion on vaccination against COVID-19 in the current scenario?

Vaccine for Corona should be taken. Publicity of it should be done and awareness should be spreaded to people.

What is your opinion on getting vaccinated against COVID-19 in the current scenario?

In past many diseases were cured due to vaccines for that disease. At present due to gods grace in India corona is not much present. It is coming under control but by vaccination complete control can be gained by us according to me.

As you said publicity should be done, according to your opinion what can we do for advertisement of vaccination?

For advertisement, there should be total ban on social media. In past fake publicity is done than case can be applied on them, in social media many misunderstandings are present, in newspaper there was a incident that in baroda a sweeper died due to heart problem which was not mentioned, instead after 2 hours of taking vaccine he died was given. So there should be specific complete ban and correct information should be given.

What is your opinion on the concern surrounding the side effects related to COVID-19 Vaccine?

According to me in past when we as a child took vaccine, fever was present as symptom so medicine was given along with it. So it is not a side effect, it states that vaccine has started working. People have developed thought that fever is side effect which is not a side effect as in body antibody is formed which results into fever than only we can come to know. Recently 2 days ago, in your medical college my friend bhagavat sinh jadeja is there who got vaccinated, he told that he had fever so due to fever we can come to know that vaccine is working. So misunderstanding should be cleared from people that fever is a not a side effect.

2. What's us your opinion on any misinformation affecting the acceptance of COVID-19 Vaccine in common people?

As I said in social medial, rumours are more spreaded than reality. Instead benefits of vaccine against corona are less present.

Scientists which are present in social media due to them more problems are created according to me. In newspaper, as corona patients interview is given like that there should be news that after vaccination of an individual no side effects are developed. Like that everyday there should be a figure of individuals who got vaccinated. False publicity which is spreaded like that good publicity should be done according to me.

Have you heard such misinformation/Rumours about COVID-19 Vaccine?

As I stated earlier, I had read news in newspaper of Divya Bhaskar that person died after vaccination but he died due to other problems and Doctors announced that he died due to other problems and not due to vaccine. Maybe it can happen that publicity is done regarding vaccination and experiments done on sick people who have got vaccinated have not developed any side effects should be given publicity according to me.

3. What are your expectations from the vaccine?

According to me corona vaccine is effective against corona and is best in India amongst the world. India is progressing in terms of it so vaccine will have its effect and is effective against corona.

What are your expectations from vaccine after vaccine is introduced?

After vaccination drive starts, except masks all precautions should be present, mask is a little bit problem other than that social distancing rule is followed and it should be implemented because corona is not abolished completely. After measles was abolished we became safe so until corona is completely abolished precautions like masks should be followed.

What do you think the government should do to meet your expectations?

Government should do publicity of it like at present mask should be weared, social distancing should be followed, frequent hand washing such precautions publicity should be done. Government should announce that precautions can be optional which should be decided by government not by personal opinion according to me.

What do you think, when you will be willing to get vaccinated?

I am ready to take vaccine today as vaccine is necessary and I am going to take vaccine and I would be first to take vaccine.

As you stated that by social distancing there are benefits present so like that what are other benefits there?

At present diseases which are responsible for corona are going to increase for that social distancing is necessary as many variaties of diseases due to virus are going to come in future so by social distancing spread of such disease can be decreased according to me.

4. At present in common people to get protection from corona, masks are wore, frequent hand washing and social distancing is present like this safe precautions are taken, after vaccination is done than according to your opinion what can be done to implement these safe precautions ?

According to me precautions like cleanliness should be maintained, so if cleanliness is maintained than not only from corona but also from other diseases we can be prevented. When an infectious person comes in contact with other person than infection like corona can be spreaded so social distancing should be followed, if mask precaution is not followed than it would be ok according to me.

After vaccination is done, for mask what is your opinion regarding it?

After vaccination is done, instantly after that mask should not be avoided, as after first dose second dose would be given than it would come to know to people that a big mass in India have got vaccinated, other types of corona can be prevailed in India so until government does not announces that we are disease free uptill than mask should be compulsory after that people according to their will they can or cannot wear mask.

5. What is your opinion on the role of COVID-19 Vaccine in ending the pandemic?

Corona vaccine is 100% working and in India government is more progressive towards vaccine as other countries are asking for our vaccines. Three companies are there and all companies have completed trials and maybe one company has not completed trials and they will complete trials. So corona vaccine has its effect and is effective.

6. Is there anything more you want to add that you were not able to say regarding acceptance and expectations of COVID-19 vaccine in this interview?

In social media there is a thought that first big people have not taken vaccine but they are not taking vaccine first because they give importance to common people, if big people are not taking vaccine than why are doctors taking vaccines so doctors are taking vaccine because they have faith in it. For eg a shop keeper is eating his own food because he has faith in his own food like that doctors are getting vaccinated because they are aware of vaccines. So if doctors are aware of vaccine so we should take vaccine as we are not doctors so I think that vaccine should be taken as vaccine is good and effective.

## **Identifier:** **Community participant 08**

**64 years old, Male, Businessman, AIK**

1. What is your opinion on vaccination against COVID-19 in the current scenario?

At present, corona vaccine introduced is a pride for our Indian nation. As we have seen diseases in past like polio, measles were there. For vaccine of these disease it took 8-10 years to be available. At present time in our nation whoever has invented the vaccine to him I would like to thank you. Almost around 8-9 months after pandemic, vaccine is available and that vaccine is demanded by almost 92 countries around the world which is a proud moment for us.

What is your opinion on getting vaccinated against COVID-19 in the current scenario?

By getting vaccinated many benefits are there like in upcoming days for our economy if corona is prevalent than it is bad for our economy. So at present if we take corona vaccine than or nation would get completely free from corona disease and compare to other countries our GDP will rise which is a possibility.

We have to take care of any types of rumours which are not spreaded. In common conditions it can be possible that if 1 lac people got vaccinated and 1 of them due to other small/large problems can get death or can fall ill for 1-2 days. In past when polio vaccination was done than this type of incidents were occurred during that time, if we ignore that fact and get corona vaccine than it is good for us.

What is your opinion on the concern surrounding the side effects related to COVID-19 Vaccine?

This is point is ridiculous like for eg if 200 vehicle are going to ahmedabad from here and 1 car got accident than 199 vehicles will not stop from going, they will keep on travelling. It can be possible that it could occur due to drivers mistake, it can occur that after taking vaccine the person is weak than it does not mean that vaccine is bad, so to get vaccinated is a good thing for everyone and vaccine should be taken by everyone.

2. What's us your opinion on any misinformation affecting the acceptance of COVID-19 Vaccine in common people?

I already stated first that rumours are spreaded all over especially in our nation, 17-18 lac people have got vaccinated from that due to other diseases 2 have died. Not more than that have died. Whoever has taken vaccine, they get common fever for around 2-4 hours or 24 hours which is common thing after getting vaccinated. Whoever is spreading rumours we have to stay alert from them and give advice to them that stop spreading such rumours because our nation is facing big loss due to it.

Have you heard such misinformation/Rumours about COVID-19 Vaccine?

This is true, at many places I have heard of rumours like by getting vaccinated we will get impotence, harm is done to body, government want us to turn towards themselves, government has other purposes by doing this. Why would government will do this to us? Government is working for us and for betterment of conmon people, government has done hard work for us. Just I saw it yesterday, For vaccine, In our budget finance minister has given different special budget for vaccine. This budget is not done free of cost, it is done for our people so for that misunderstanding which has occurred in our minds should be prohibited. In past whichever incidents have occurred due to vaccination which I also have suffered like measles. At present our nation is polio free, we are completely free from measles like that many diseases were there from which we are freed. Like that in upcoming days, to get free from corona than everyone has to take corona vaccine compulsory and it is for our betterment.

As we dicussed regarding misunderstandings which are there. What can we do to stop spreading rumours?

For that young people, team of doctors or medical staff is there, people who are running NGOs, big organisations. Like in recent time Nanakbhai Bhatt from sishuvihar had a talk with me that preparations should be done over here for vaccination so that we can get people vaccinated. By social media, newspaper, letters, Government Mp, MLA or which ever persons are there or corporaters are there. If All of this people unite and face these rumours than according to me in upcoming days in our nation corona will be removed. At present yesterday in Bhavnagar 00 cases are present than in upcoming days if we all get vaccinated than in our nation there would be 00 cases so our economy will also get boosted.

3. What are your expectations from the vaccine?

UpTill now government has done whatever they can do but in every aspects government is unable to reach. So permission should be given to private doctors or hospitals to start vaccination. From government, vaccination is done free of cost. In private hospitals if vaccination is done at cost of Rs 1000,1500 or 2000 than people can afford it. People will get vaccinated by spending money. Those families who are rich, they get their child vaccinated by spending money, common people would get their child vaccinated from Red Cross society, get vaccinated from government hospital also. If such freedom is given than vaccine will reach people fastly, we should try regarding it.

What do you think the government should do to meet your expectations?

From government as I stated earlier if permission is given to private doctor, people who have recently joined government medical store by them vaccine is given from home to home vaccination is done. Especially people who are above 50 years, government has already done announcement that first police department, medical staff, cleaning workers are there to them first vaccine will be given and recently our Gujarat’s chief minister has announced that first of all they would get completely vaccinated and after that employees which are in government will get vaccinated.

What do you think, when you will be willing to get vaccinated?

Whenever I will be asked for vaccination I am ready for that and I have already talked to them that whenever vaccination is started I am ready for that and almost I have sent this message to 700-800 people like some news channel has taken interview of mine I have done appeal of it and my social media friends have taken interview like this from me and I have done appeal to take vaccine and it is for our betterment and those people who are leaders of community if they come forward to it than people will get good message and get ready to get vaccinated. Those benefits will help nation to go forward according to me.

4. At present in common people to get protection from corona, masks are wore, frequent hand washing and social distancing is present like this safe precautions are taken, after vaccination is done than according to your opinion what can be done to implement these safe precautions ?

At present WHO has given guideline and from health department guidelines are given regarding it vaccination is done which is a good thing. But along with it for safety to wear mask, frequent hand washing, social distance should be maintained is necessary. Uptill when whole disease comes under our control vaccination those precautions are necessary. This thing is for our benefit like in future it should not happen that vaccination is running and this disease is progressing so for that if we will be irresponsible and social distancing is needed to be followed because it is necessary.

5. What is your opinion on the role of COVID-19 Vaccine in ending the pandemic?

In whole world, especially this pandemic is affected in Europe and other countries, lockdown is still present. And at present 99% of our nation is unlocked and our economy is improving. So by vaccination at present our country has benefit but in some other countries due to irresponsiblity repeatedly lockdown is announced and death rate is higher and corona cases are higher and in our country it is not there. Our nations people are having more understanding and by that safety is present which is also necessary.

6. Is there anything more you want to add that you were not able to say regarding acceptance and expectations of COVID-19 vaccine in this interview?

No, most of things I have already stated and for vaccination more and more people come and by themselves they come for vaccine registration like if main member of a home gets vaccinated than other members will get ready. Many times other members will not get vaccinated due to fear of corona virus from last 9-10 months, the reality is deaths from fear of corona are more than the people suffering from corona and like that deaths from cancer are more present. Corona is an infectious disease so people have fear in their mind due to it. I personally took people when death were peevalent who had fear of corona to burial sites , to 40-45 people who have died due to corona I have done personally burial rituals. At that time fear atmosphere was there in people which is still present like if anyone has got corona than that individual is considered to be insignificant so if main member of a family takes vaccine and nothing happens to them than there would be a happy atmosphere in home than other memebers and people of another house will get ready to get vaccinated.

Thank you for taking participation in this activity. Thank you sir.

Thank you.

## **Identifier: Community participant 09**

**72 years old, Male, Retired, HA**

1. What is your opinion on vaccination against COVID-19 in the current scenario?

I am not going to take vaccine.

Any reasons why are you not going to take vaccine?

There is no such type of thing as vaccine so it is not needed.

What is your opinion on the concern surrounding the side effects related to COVID-19 Vaccine?

If side effects are there than where would people go, injection is given and than you go rather

you die or live what would it mean to us so I am not going to take it.

What's us your opinion on any misinformation affecting the acceptance of COVID-19 Vaccine in common people?

I am not listening to such rumours among people as I am not having talk regarding it with

people.

2. What are your expectations from the vaccine?

When vaccine will come everything will become normal, at present side effects are there, I am not having any person to take care of mine, I am alone so I do not want to die at present due to it.

3. What do you think the government should do to meet your expectations?

That is not my topic to discuss about it.

What do you think, when you will be willing to get vaccinated?

Not at present but in future I will think about it.

At present in common people to get protection from corona, masks are wore, like this safe

precautions are taken, after vaccination is done than according to your opinion what can be done to implement these safe precautions?

After all have taken vaccines and disease would get vanished than there is no need to wear it.

4. What is your opinion on the role of COVID-19 Vaccine in ending the pandemic?

No idea.

5. Is there anything more you want to add anything regarding it?

No.

Thank you.

## **Identifier: Community participant 10**

**77 years old, Male, Retired, AR**

1. What is your opinion on vaccination against COVID-19 in the current scenario?

There is no meaning of it. I do not want to say anything about it.

What is your opinion on getting vaccinated against COVID-19 in the current scenario?

I am not willing to take vaccine.

What are the reasons for not acceptance of Vaccine?

I do not feel any need about it.

What is your opinion on the concern surrounding the side effects related to COVID-19 Vaccine?

It can occurs, side effects can occur. Yes I have heard about it but I do not have taken any interest towards it.

What's us your opinion on any misinformation affecting the acceptance of COVID-19 Vaccine in common people?

Two aspects are there, it can be true or false. I am not going to take vaccine.

2. What are your expectations from the vaccine?

It should not be taken.

3. What do you think the government should do to meet your expectations?

How can we tell until it gets confirmed.

Can you elaborate regarding how it gets confirmed?

No.

What do you think, when you will be willing to get vaccinated?

Not at all.

At present in common people to get protection from corona, masks are wore, like this safe precautions are taken, after vaccination is done than according to your opinion what can be done to implement these safe precautions ?

Uptill it is necessary than we have to do it like wearing masks and using sanitizer.

4. What is your opinion on the role of COVID-19 Vaccine in ending the pandemic?

We do not feel any role of it in ending it.

5. Is there anything more you want to add that you were not able to say?

No I do not want to say anything.

Thank you.

## **Identifier:** **Community participant 11**

**66 years old, Male, Auto rickshaw driver, AD**

1. What is your opinion on vaccination against COVID-19 in the current scenario?

Two aspects are there: yes or no. My mind is already saying no for it

What are the reasons for your not acceptance of Vaccine?

Due to all the rumours present, I have fear due to it.

Which type of rumours have you heard of?

Like government is planning to kill everyone like that type of different mouth rumours I have heard of and I am not having interest in getting vaccinated.

What is your opinion on the concern surrounding the side effects related to COVID-19 Vaccine?

People are in fearful state of mind as there are two aspects so people would not get vaccine so I am not having trust in it.

What's us your opinion on any other rumours which you would like to add regarding COVID-19 Vaccine among people?

Various rumours as people wants to live, they do not want to die by taking vaccine like various spare parts of body are affected like kidney are affected so.

2. What are your expectations from the vaccine?

I do not have experience regarding it as I have only heard that due to rumours people are in fearful state of mind, as in present people are in fear of corona so they are wearing masks, to do as government says all that things are still going on like washing hands, social distancing, wear masks until that period of time we will follow till government states us otherwise no one has trust in vaccination as all are spreading rumours so I do not have interest in it.

3. What do you think the government should do to meet your expectations?

I do not have experience of it whatever government has to do regarding it I do not have any experience.

What do you think, when you will be willing to get vaccinated?

When my family doctor will say, my family, my community will have trust and when they say that it is needed to be taken at that time if it is necessary than I am ready for it.

At present in common people to get protection from corona, masks are wore, frequent hand washing is present like this safe precautions are taken, after vaccination is done than according to your opinion what can be done to implement these safe precautions?

After that no question arises, after that vaccination is not needed, if any new illness arises new medicine would come that it would be done otherwise at present none of my family member is ill due to gods will. That’s all.

4. What is your opinion on the role of COVID-19 Vaccine in ending the pandemic?

If it becomes successful than it is good as people will get fearless. If it goes successful than people will have faith in others although after 6 months, people will get fearless.

People are fearful due to corona and it’s vaccine so what can we do to remove fear from them?

That I don’t know. I don’t have that much knowledge regarding it. I told you things regarding it according to my capacity, cleanliness should be maintained, talking should be reduced, if any type of cough or cold develops than instantly take medicines like that.

Which type of vaccine should be introduced so that people would get vaccinated?

It is of natural type, if they have trust on themselves than they would have medicine and prayers both, both medicines and prayers should be continued.

5. Is there anything more you want to add regarding COVID-19 vaccine in this interview?

No.

Thank you.

## **Identifier: Community participant 12**

**55 years old, Male, Employee, JB**

1. What is your opinion on vaccination against COVID-19 in the current scenario?

Whichever information we are getting from social media or television from that we would think that India’s vaccine has not completed all tests from which third phase trial is not yet completed from the report which obviously we do not know, they would know but if it would be completed than it would be good. Vaccine should be present it is a good aspect and though it is not the first vaccine introduced like in past bcg vaccines were introduced by many countries government at that time they would have done enough research though side effects may be present so that vaccine might be more successful than this type according to me. In past vaccines like Bcg, polio vaccine by government and private sectors, vaccine is given by physicians and charts were prepared for it. So more research was necessary for this (corona) vaccine.

What is your opinion on getting vaccinated against COVID-19 in the current scenario?

This vaccine should be optional, those who want to get vaccinated should get it which is current scenario. So those who want to get vaccinated they should get it and those who don’t would not get it.

What is your opinion on the concern surrounding the side effects related to COVID-19 Vaccine?

Today there is an article given in Gujarat samachar that a cleaner worker named Jignesh was given vaccine yesterday or day before yesterday than after 2-2.5 hrs he died which is a big article in today’s newspaper. Their house members stated that he died due to vaccine, if didn’t took vaccine than he wouldn’t die and in opposition there was a panel of doctors and they stated that other reasons were there for death. So this type of mix things are present but this is present in today’s newspaper.

What's us your opinion on any misinformation COVID-19 Vaccine ?

To remove such rumours best solution is that prime minister, president, Vice President, and central government head should take vaccine first by showing to people that they took vaccine by taking out from packet and got vaccinated in front of all people. All big leaders have not got vaccinated and they are telling people to get vaccinated. In that aspect you can see that prime ministers of china and Russia have got vaccinated and over here they have not taken and people are doing publicity of it over here.

Have you heard such misinformation/Rumours about COVID-19 Vaccine?

How can we say rumor that it is wrong as In today’s article of Gujarat samachar. So there is a mix trends of it is present so we cannot state it completely wrong, whatever wrong or right if happened with us than only we can state it. Like it comes in every newspaper, social media, tv news channel than how can tell that it is wrong. As in today’s newspaper how can an ordinary man can decide that it is a rumor or true talk.

2. What are your expectations from the vaccine?

It should be effective and if possible should cover all age groups and if possible due to its good results after 3-5 months corona gets eliminated than it is good.

3. What do you think the government should do to meet your expectations?

Wide publicity,awareness campaign, as I told you that any celebrities and leaders take vaccine than there will be more benefit so people will get motivation that they should get vaccinated.

What do you think, when you will be willing to get vaccinated?

Not at present. For that I will give the reason that I do not feel personally that I need it and it will cure me. Though having vaccine there is a decline in cases like in Gujarat, in Bhavnagar than it will be ok if I don’t get vaccine. If there would be a new strain introduced than new problems arises and there would be a rise in number of cases. There was a projection by panel of doctors of AIIMS which I was listening that in feb/march there would be a great decrease in number of cases which is already been seen 2-4 months before that there would be a declining trend in number of cases. At present we do not know increase/decrease in number of testing that actuall people have been cured. But apparently it has decreased a lot so in that conditions if vaccine would be optional than it will be ok.

As you stated about awareness campaign, so what can be done to increase awareness among common people?

Neutral people like some NGOs if give wide publicity than that would be better. Other than that if government medium would give publicity than people would thank that it is for their publicity and for their goodness. If NGOs, or doctors like you who are studying if such people give awareness than it would create a difference in mindset of people.

At present in common people to get protection from corona, masks are wore,like this safe precautions are taken, after vaccination is done than according to your opinion what can be done to implement these safe precautions ?

I am in a little doubt regarding it. For that doctors, researchers can say regarding it, as a common man how can we say about it. For that instead of asking people, what are government guidelines or by any research or establishment should be done than after that they should declare that those who have taken vaccine they didn’t need to wear mask or use of sanitizer is not necessary. It should be placed at a research level according to my opinion.

4. What is your opinion on the role of COVID-19 Vaccine in ending the pandemic?

It can be good and as results will come we can know about it. But there is a thing that pandemic has decreased by its own as you can observe it.

5. Is there anything more you want to add that you were not able to say regarding acceptance and expectations of COVID-19 vaccine in this interview?

No, this is all the things that I would like to share with you.

Thankyou.
