# Supplementary material for: Acceptance and expectations of healthcare workers and community during the COVID-19 vaccine rollout in Bhavnagar city, western India: a qualitative exploration
Source: BMC Health Serv Res. 2024 Mar 27;24:386. doi: 10.1186/s12913-024-10885-5 (PMC10976747; doi:10.1186/s12913-024-10885-5)
Supplement: Supplementary file 3 — Supplementary Material 3. [file 12913_2024_10885_MOESM3_ESM.docx]

**Codebook**

**Perceptions of healthcare workers and community regarding acceptance and expectation from COVID-19 vaccine in Bhavnagar during January 2021**

| **Theme** | **Subtheme** | **Category** | **Codes** | **Explanation** |
| --- | --- | --- | --- | --- |
| **Willingness** | Perceived Susceptibility | COVID phobia | Impending COVID-19 infection | Fear of COVID infection |
|  |  |  | No specific treatment | Unavailability of Medicines against COVID19 |
|  | Perceived Severity | Fear | Hospitalization | Fear of staying at hospital for treatment |
|  |  |  | Death or disability | Afraid of death or disability due to COVID19 |
|  |  |  | Social Isolation | Getting freedom from Home isolation for days to weeks |
|  | Perceived Benefits | Halt pandemic | Avoid lockdown | Prevent the impact of lockdown on personal, familial and Social life |
|  |  |  | Prevent another wave | Prevent possible future wave |
|  |  | Protection | Protection of self | Less chance of getting covid infection |
|  |  |  | Protection of family | Protect family from getting infected with covid19 |
|  |  |  | Financial protection | Improvement in National Economy and individual financial condition |
|  | Perceived Barriers | Vaccine availability | Availability of vaccine | Enough vaccination centers and doses for all |
|  | Cue to Action | Trust | Clinical trials data | Trust in Clinical trials conducted |
|  |  |  | Faith in government | Trust in Government-provided vaccine |
|  |  |  | Doctors' advice | Trust in doctors who have got vaccinated and suggesting vaccine |
|  |  |  | Established Vaccine safety | Safe and successful vaccination accomplished in HCWs |
|  |  | Moral Duty | Positively influence others | To lead by example to motivate others to take vaccine |
|  |  |  | Duty as a person | Moral duty as a person and as an Indian citizen |
|  |  |  | Controlling Spread of Misinformation | Controlling Spread of Misinformation in news or social media |
|  |  |  | Awareness by more IEC | More IEC from government to increase awareness and address misinformation |
| **Refusal** | Perceived Susceptibility | Natural protection | Not infected till date | Not infected with covid19 despite so many covid19 cases in community |
|  |  |  | Already exposed to covid19 | Already working among covid19 patients for months so immune against it. |
|  |  |  | Not a real disease | Perception that covid is not a real disease, |
|  | Perceived Severity | Milder severity | Not a severe disease | Belief that Covid19 is not a severe disease as most of infected people have recovered |
|  |  |  | No fear of covid19 | Not afraid of covid19 infection at all |
|  | Perceived Benefits | Questionable need | No benefit from vaccine | Belief that covid vaccine do not provide protection |
|  |  |  | No need of vaccine | Belief that they do not require any vaccine |
|  |  |  | Declining covid19 cases | No need of vaccine as already covid cases are declining |
|  | Perceived Barriers | Fear | Long term side effects | Anxious about possible long term side effects of vaccine |
|  |  |  | Sterility | Afraid that vaccine will cause sterility |
|  |  |  | Death or disability | Afraid of death, disability or serious side effects due to vaccine |
|  |  |  | New vaccine | Afraid of taking new vaccine |
|  |  |  | Covid infection due to vaccine | Perception that people getting infected after taking vaccine |
|  |  | Concerns | Transparency | No transparency in sharing clinical trials data and AEFI data with public |
|  |  |  | Rapid development of vaccine | Covid vaccine is developed in record breaking time raising concerns regarding its efficacy |
|  |  |  | Duration of protection | How long will immunity last after taking vaccine? |
|  |  |  | Protection from new strains | Whether existing vaccine will provide protection against new strains or not? |
|  |  |  | Family responsibility | Who will be responsible for their family if anything happens to them |
|  |  |  | Wait and watch | Wait and watch vaccination in other people and will decide after some time. |
|  |  | News Media | Rumors | Misinformation flooded in newspapers, news media and social media |
|  |  |  | Hearsay | Information gained from others and not part of their direct knowledge |
|  |  |  | Unaccountable media reports | Irresponsible reporting without verifying actual truth and selective emphasis of negative news |
|  |  |  | Lack of awareness | Lack of awareness about vaccine and benefits of vaccine |
|  |  |  | Infodemic | So much information in social media and news, can't decide between rumors and truth |
|  |  | Lack of faith | Government-provided vaccine | Lack of trust in government-provided vaccine |
|  |  |  | Conspiracy | Belief that covid vaccine is for political agenda and financial gain and not for benefits of public |
|  |  |  | Clinical trial's robustness | Lack of trust in clinical trails conducted for vaccine |
|  |  |  | God will protect | Belief of protection from their divine force more than vaccine |
|  | Cue to Action | Influence | Community Influence | Opinions derived from Community belief |
|  |  |  | Religious leaders | Influence of religious leaders |
| **Expectations** |  | From vaccine | Safe vaccine | No or least side effects from vaccine |
|  |  |  | Maximum efficacy | 100% protection from COVID after vaccination |
|  |  |  | Longer duration of protection | Long lasting immunity against COVID19 |
|  |  |  | Effective against multiple strains | Protection from future new strains of COVID19 |
|  |  |  | Non-parenteral route of vaccine | oral or nasal route for vaccine delivery |
|  |  | From government | Free of cost | Should be provide free of cost |
|  |  |  | Vaccine for all ages | Vaccine should be made available for all age groups |
|  |  |  | Rapid and wide coverage | Covering major population within shorter duration |
|  |  |  | Vaccination in private hospital | Involvement of Private health sector for rapid coverage |
|  |  |  | Grievance redressal system | Effectively working helpline numbers to address the grievance |
|  |  |  | AEFI management | Preparedness for possible minor to major AEFI at vaccination centers |
|  |  |  | Compensation for AEFI | Monetary compensation for AEFI as per severity |
|  |  |  | Awareness campaigns | Increase Awareness campaigns using all possible modes of communications |
|  |  |  | Action against miscreants | Strict actions against people spreading rumors and misinformation |
|  |  |  | Vaccinating leaders first | Vaccination of Political, Religious and community leaders first to build confidence |
|  |  |  | Enabling vaccine production | Protections of vaccine producing pharmaceuticals against unnecessary lawsuits |
|  |  |  | Transparency | Transparency in sharing full result of clinical trials conducted |
|  |  | Post vaccination | Ending pandemic | Successful vaccination leading to herd immunity which ends pandemic |
|  |  |  | Freedom from mask | Life free from Mask in daily life |
|  |  |  | Back to pre-COVID era | Getting back to restrictions free Precovid life |
|  |  |  | Boost in economy | Lifting up the national economy |
| **Challenging factors** |  | Human resources | Less human resources | Limited numbers of HCWs |
|  |  |  | Overburden | Simultaneously running covid vaccination with other health programs |
|  |  | Use of information & technology | Data entry issue | Data entry and verification issue |
|  |  |  | Internet connectivity | Lack/Slow of internet connectivity |
|  |  |  | Slow server | Slow COWIN Server with Multiple users pan India |
|  |  | Community behaviors | Misbelief | Community misbelief regarding vaccine |
|  |  |  | Influence of negative news | Rumors and misinformation spreading fast than IEC |
|  |  |  | Conspiracy theories | Perception that vaccine is meant to cause harm |
|  |  |  | Resistant community | Resistant community which shows refusal for all vaccines |
|  |  |  | Fear of vaccine | Fear of vaccine side effects |
| **Enabling factors** |  | Established universal immunization programs | Trained staffs in AEFI management | staff well prepared and trained for management of possible AEFI |
|  |  |  | Oriented with vaccination program | staff well experienced with vaccination program due to UIP |
|  |  | Grievance redressal system | COVID helpline | Helpline number to address public issues |
|  |  |  | Aarogya setu app | Help desk setups |
|  |  |  | Preparedness for AEFI management | AEFI management centers and corners established with AEFI management training of staff |
|  |  | Use of information & technology | CoWIN portal and app | Digitalization of Vaccination for easy Data management |
|  |  |  | Easy registration & verification process | Use of IT made registration and verification easy |
|  |  |  | SMS reminders | Convenient method for communication with vaccine beneficiaries for 2nd dose |
|  |  | Logistics | Available logistics | Enough logistics supply in advance |
|  |  | Private health sector | Involvement of private sector | Participation of Private health sector for vaccination and creating awareness |
