# Supplementary material for: Acceptance and expectations of healthcare workers and community during the COVID-19 vaccine rollout in Bhavnagar city, western India: a qualitative exploration
Source: BMC Health Serv Res. 2024 Mar 27;24:386. doi: 10.1186/s12913-024-10885-5 (PMC10976747; doi:10.1186/s12913-024-10885-5)
Supplement: Supplementary file 4 — Supplementary Material 4. [file 12913_2024_10885_MOESM4_ESM.docx]

Study title: “Acceptance and expectations of healthcare workers and community during the COVID-19 vaccine rollout in Bhavnagar city, western India: a qualitative exploration”

COREQ (COnsolidated criteria for REporting Qualitative research) Checklist

A checklist of items that should be included in reports of qualitative research. You must report the page number in your manuscript where you consider each of the items listed in this checklist. If you have not included this information, either revise your manuscript accordingly before submitting or note N/A.

| **Topic** | **Item No.** | **Guide Questions/Description** | **Reported on**  **Page No.** |
| --- | --- | --- | --- |
| **Domain 1: Research team and reﬂexivity** | | | |
| *Personal characteristics* | | | |
| Interviewer/facilitator | 1 | Which author/s conducted the interview or focus group? | Page No. 6,  Methods, Research Team |
| Credentials | 2 | What were the researcher’s credentials? E.g. PhD, MD | Page No. 6,  Methods, Research Team |
| Occupation | 3 | What was their occupation at the time of the study? | Page No. 6,  Methods, Research Team |
| Gender | 4 | Was the researcher male or female? | Page No. 6,  Methods, Research Team |
| Experience and training | 5 | What experience or training did the researcher have? | Page No. 6,  Methods, Research Team |
| *Relationship with participants* | | | |
| Relationship established | 6 | Was a relationship established prior to study commencement? | Page No. 9,  Methods, Data collection |
| Participant knowledge of  the interviewer | 7 | What did the participants know about the researcher? e.g. personal goals, reasons for doing the research | Page No. 6,  Methods, Research Team |
| Interviewer characteristics | 8 | What characteristics were reported about the inter viewer/facilitator?  e.g. Bias, assumptions, reasons and interests in the research topic | Page No. 7,  Methods, Study setting |
| **Domain 2: Study design** | | | |
| *Theoretical framework* | | | |
| Methodological orientation and Theory | 9 | What methodological orientation was stated to underpin the study? e.g. grounded theory, discourse analysis, ethnography, phenomenology, content analysis | Page No. 5 and 10,  Methods, Theoretical framework and Analysis |
| *Participant selection* | | | |
| Sampling | 10 | How were participants selected? e.g. purposive, convenience,  consecutive, snowball | Page No. 8,  Methods, Study population and sampling |
| Method of approach | 11 | How were participants approached? e.g. face-to-face, telephone, mail,  email | Page No. 9,  Methods, Data collection, 2^nd^ Paragraph |
| Sample size | 12 | How many participants were in the study? | Page No. 9,  Methods, Data collection, 3^rd^ Paragraph |
| Non-participation | 13 | How many people refused to participate or dropped out? Reasons? | Page No. 10,  Results, 1^st^ Paragraph |
| *Setting* | | | |
| Setting of data collection | 14 | Where was the data collected? e.g. home, clinic, workplace | Page No. 9,  Methods, Data collection, 2^nd^ Paragraph |
| Presence of non-participants | 15 | Was anyone else present besides the participants and researchers? | Page No. 9,  Methods, Data collection, 2^nd^ Paragraph |
| Description of sample | 16 | What are the important characteristics of the sample? e.g. demographic data, date | Page No. 8,  Methods, Study population and sampling |
| *Data collection* | | | |
| Interview guide | 17 | Were questions, prompts, guides provided by the authors? Was it pilot tested? | Yes. Page No. 9,  Methods, Data collection, 1^st^ Paragraph and  Additional File No. 1 |
| Repeat interviews | 18 | Were repeat inter views carried out? If yes, how many? | No. Page No. 10,  Methods, Data collection, 4^th^ Paragraph |
| Audio/visual recording | 19 | Did the research use audio or visual recording to collect the data? | Yes. Page No. 10,  Methods, Data collection, 4^th^ Paragraph |
| Field notes | 20 | Were field notes made during and/or after the interview or focus group? | Yes. Page No. 9,  Methods, Data collection, 3^rd^ Paragraph |
| Duration | 21 | What was the duration of the inter views or focus group? | Page No. 9,  Methods, Data collection, 3^rd^ Paragraph |
| Data saturation | 22 | Was data saturation discussed? | Yes. Page No. 9,  Methods, Data collection, 3^rd^ Paragraph |
| Transcripts returned | 23 | Were transcripts returned to participants for comment and/or correction? | No. Page No. 10,  Methods, Data collection, 4^th^ Paragraph |
| **Domain 3: analysis and ﬁndings** | | | |
| *Data analysis* | | | |
| Number of data coders | 24 | How many data coders coded the data? | Two data coders.  Page No. 10, Methods, Analysis |
| Description of the coding  tree | 25 | Did authors provide a description of the coding tree? | Yes.  Additional File No. 3 |
| Derivation of themes | 26 | Were themes identified in advance or derived from the data? | Identified in advance.  Page No. 10, Methods, Analysis |
| Software | 27 | What software, if applicable, was used to manage the data? | Microsoft word.  Page No. 10, Methods, Analysis |
| Participant checking | 28 | Did participants provide feedback on the findings? | No.  Page No. 10, Methods, Data collection, 4^th^ Paragraph |
| *Reporting* | | | |
| Quotations presented | 29 | Were participant quotations presented to illustrate the themes/findings?  Was each quotation identified? e.g. participant number | Yes.  Page No. 12-24, Results.  Yes.  Page No. 12-24, Results. |
| Data and findings consistent | 30 | Was there consistency between the data presented and the findings? | Yes.  Page No. 12-25, Results. |
| Clarity of major themes | 31 | Were major themes clearly presented in the findings? | Yes.  Page No. 12-24, Results. |
| Clarity of minor themes | 32 | Is there a description of diverse cases or discussion of minor themes? | Yes.  Page no. 25, results. |

Developed from: Tong A, Sainsbury P, Craig J. Consolidated criteria for reporting qualitative research (COREQ): a 32-item checklist for interviews and focus groups. *International Journal for Quality in Health Care*. 2007. Volume 19, Number 6: pp. 349 – 357
